# *In situ* Stiffness Adjustment of AFM Probes by Two Orders of Magnitude

**DOI:** 10.3390/s16040523

**Published:** 2016-04-12

**Authors:** Marcel Lambertus Cornelis de Laat, Héctor Hugo Pérez Garza, Murali Krishna Ghatkesar

**Affiliations:** 1 Department of Precision and Microsystems Engineering (PME), Faculty of Mechanical, Maritime and Materials Engineering (3mE), Delft University of Technology, Mekelweg 2, 2628 CD Delft, The Netherlands; m.l.c.delaat@student.tudelft.nl; 2 DENSsolutions BV, Informaticalaan 12, 2628ZD Delft, The Netherlands; hugo.perez@denssolutions.com

**Keywords:** stiffness, adjustable stiffness, stiffness tuning, AFM, Atomic Force Microscope

## Abstract

The choice on which type of cantilever to use for Atomic Force Microscopy (AFM) depends on the type of the experiment being done. Typically, the cantilever has to be exchanged when a different stiffness is required and the entire alignment has to be repeated. In the present work, a method to adjust the stiffness *in situ* of a commercial AFM cantilever is developed. The adjustment is achieved by changing the effective length of the cantilever by electrostatic pull-in. By applying a voltage between the cantilever and an electrode (with an insulating layer at the point of contact), the cantilever snaps to the electrode, reducing the cantilever’s effective length. An analytical model was developed to find the pull-in voltage of the system. Subsequently, a finite element model was developed to study the pull-in behavior. The working principle of this concept is demonstrated with a proof-of-concept experiment. The electrode was positioned close to the cantilever by using a robotic nanomanipulator. To confirm the change in stiffness, the fundamental resonance frequency of the cantilever was measured for varying electrode positions. The results match with the theoretical expectations. The stiffness was adjusted *in situ* in the range of 0.2 N/m to 27 N/m, covering two orders of magnitude in one single cantilever. This proof-of-concept is the first step towards a micro fabricated prototype, that integrates the electrode positioning system and cantilever that can be used for actual AFM experiments.

## 1. Introduction

The Atomic Force Microscope (AFM) was invented by Binnig, Quate and Gerber in 1986 [1]. AFM is a technology that can image samples with atomic resolution [2]. An AFM uses a silicon based chip with a cantilever beam and a very sharp tip to probe a surface. The forces that act on the tip cause the cantilever beam to deflect, which is measured with a sensor. With this information, the topography of the surface can be reconstructed. It is also possible to calculate the force between tip and sample by using the (known) stiffness of the cantilever by using Hooke’s law. This information can be used to measure material properties like Young’s modulus [3] and molecular interaction forces [4,5].

**Figure 1:**
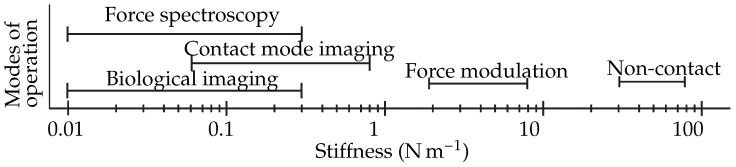
The range of stiffness for the AFM cantilever for different imaging modes.

The stiffness of the cantilever is very important to the type of measurement that needs to be performed; for a different type of measurement a different stiffness is needed [6]. The range of cantilever stiffness for different type of measurements is shown Figure 1. In practice, users have to exchange the cantilever if they want a different stiffness; this consumes a lot of time. Furthermore, the alignment of the sensor has to be repeated and the region of interest needs to be reidentified. It would provide a great advantage if the stiffness of an AFM probe could be changed *in situ*. An example of a potential application is Peak Force® Tapping mode, which is used for simultaneous imaging and Young’s modulus mapping [7]. The stiffness of the cantilever should match the Young’s modulus for high accuracy measurements. Samples with high Young’s modulus variability cannot be accurately measured with a fixed stiffness. An adjustable stiffness probe would provide the solution to successfully measure such samples. Another example would be to combine force spectroscopy [8], which requires a soft cantilever with non-contact mode imaging [9] which requires a stiff cantilever. In principle, any mechanical characterization of an unknown sample require knowledge and/or optimization of the cantilever spring constant. Therefore, stiffness adjustment will be useful for variety of operating modes like QI™mode, HybriD Mode™, Force Volume mode, *etc*.

Earlier attempts to design an *in situ* tunable stiffness probe have been made. Mueller-Falcke *et al.* designed a switchable stiffness probe [10,11]. This probe can switch between 0.01 N/m and 0.1 N/m. This change in stiffness is sufficiently large to switch to a different AFM mode, but the probe is not compatible with conventional AFM systems. This probe does not use a cantilever beam, but an in-plane moving shuttle. In order to do AFM measurements with this probe, the system must be adapted to this type of probe. Miguel V. Vitorino *et al.* reported a resonance tuning of micro and nano-metric cantilevers by using proportional and differential gains of the oscillation feedback control [12]. For one of the microcantilevers with stiffness of 0.01 N/m, resonance frequency tuning of about one order in magnitude was reported. No stiffness values were calculated. Kawai *et al.* presented an AFM probe of which the stiffness can be tuned by changing the second moment of inertia [13]. A piezoelectric actuator can change the curvature of this cantilever. It is compatible with conventional AFM systems. The proven change in stiffness is only 14%, which is too small to switch to another mode. An AFM probe with *in situ* adjustable stiffness, which is both compatible with conventional AFM systems and has a large tuning range is presented in this paper. Results from analytical, finite element methods and experiments are presented.

**Figure 2:**
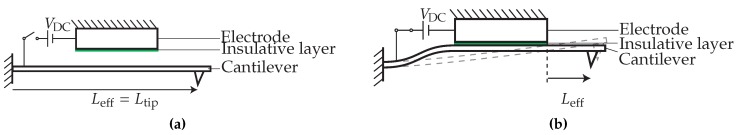
In (**a**) the cantilever is in unadjusted state. When the bias voltage $V_{\mathrm{DC}}$ is applied, the cantilever will start bending towards the electrode; When the bias voltage is bigger than the pull-in voltage, the cantilever will pull-in towards the electrode (**b**). The cantilever will hit the electrode (dashed line) and will be in a stable state. When the voltage is further increased, the cantilever will finally be in the adjusted state. The effective length $L_{\mathrm{eff}}$ of the cantilever has decreased, and the stiffness is increased.

The change in stiffness is achieved by reducing the effective length of the cantilever. This is done by placing a parallel electrode close to the cantilever and applying a bias voltage $V_{\mathrm{DC}}$ between the cantilever and this electrode as shown in Figure 2. The cantilever pulls-in to the electrode (dashed line). The insulator ensures no electrical contact between the cantilever and electrode. When the voltage is further increased, the cantilever will end up in the final clamped state (solid line). The cantilever is clamped to the electrode due to the electrostatic force. The change in effective length of the cantilever results in a change in stiffness. The stiffness of a cantilever beam is governed by the following equation:

(1) $$\begin{array}{c} k_{\mathrm{eff}}=\frac{3EI}{L_{\mathrm{eff}}^{3}} \end{array}$$

where *E* is the Young’s modulus, *I* the second moment of inertia and $L_{\mathrm{eff}}$ the effective length. So if the effective length is reduced, the stiffness will increase. For example; in order to increase the stiffness one order of magnitude, the effective length should decrease $\sqrt[{3}]{10}=2.15$ times. Note that for accurate measurement of stiffness, Sader method is recommended which is based on thermal resonance frequency spectrum [14,15].

## 2. Modeling

### 2.1. Analytical Model

When a voltage is applied, the cantilever will start bending towards the electrode. The mechanical restoring forces will increase linearly with displacement, while the electrostatic force increases inversely quadratic with displacement. When the electrostatic force is larger than the mechanical restoring force, pull-in will occur. This corresponds with the state which is indicated with the dashed line in Figure 2. A model is developed to determine the required voltage to achieve electrostatic pull-in. The model is based on Do *et al.* [16] and is modified such that it can be used for this particular system. The derivation of this model and experimental verification are found in the supplementary material. The required bias voltage $V_{\mathrm{DC}}$ for equilibrium is expressed as:

(2) $$V_{\mathrm{DC}}=\sqrt{\frac{12\hat{E}Iy\left(L_{\mathrm{cel}}\right)}{\epsilon_{\mathrm{air}}\epsilon_{\mathrm{ins}}^{2}w_{\mathrm{eff}}\int_{L_{\mathrm{cel}}-\alpha L_{\mathrm{cel}}}^{L_{\mathrm{cel}}}\frac{x^{2}\left(3L_{\mathrm{cel}}-x\right)}{{\left(\epsilon_{\mathrm{air}}d_{\mathrm{ins}}+\epsilon_{\mathrm{ins}}\left(g_{0}-y\left(x\right)\right)\right)}^{2}}dx}}$$

**Figure 3:**
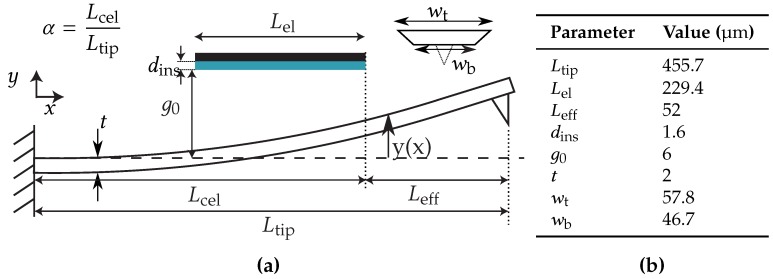
Schematic with the corresponding (**a**) geometry of the finite element model and (**b**) parameters used.

The pull-in voltage ($V_{\mathrm{PI}}$) is found when $V_{\mathrm{DC}}$ is at its maximum. For voltages higher than $V_{\mathrm{PI}}$, the system is unstable. The geometric parameters are shown in Figure 3a. $\hat{E}$ is the effective Young’s modulus ($\hat{E}=E/\left(1-\nu^{2}\right)$ with *E* and *ν* the Young’s modulus and Poisson’s ratio respectively), *I* the second moment of inertia, $g_{0}$ the initial distance between the electrode and cantilever, $\epsilon_{\mathrm{air}}$ and $\epsilon_{\mathrm{ins}}$ are the dielectric permittivity in air and insulator respectively. The effective width of the cantilever $w_{\mathrm{eff}}$ compensates for fringe field effects [17]:

(3) $$\begin{array}{c} w_{\mathrm{eff}}=w\left(1+0.65\frac{\left(1-y\left(L_{\mathrm{tip}}\right)/g_{0}\right)g_{0}}{w}\right) \end{array}$$

In order to be able to calculate the electrostatic force, an assumption is made on the shape of the cantilever y(x), which is fitted to FEM results and is found to be:

(4) $$y\left(x\right)=\left(2.56-\frac{16.127}{4{\left(x/L_{\mathrm{tip}}+0.00185\right)}^{2}+6.2786}\right)y\left(L_{\mathrm{tip}}\right)$$

The deflection at the tip $y(L_{\mathrm{tip}})$ is found by linearly extrapolating the deflection at $y(L_{\mathrm{cel}})$:

(5) $$y\left(L_{\mathrm{tip}}\right)=y\left(L_{\mathrm{cel}}\right)+\frac{\;dy(x)}{\;dx}{|}_{L_{\mathrm{cel}}}\left(L_{\mathrm{tip}}-L_{\mathrm{cel}}\right)$$

### 2.2. FEM Model

A finite element model was developed in COMSOL Multiphysics${}^{®}$ for comparison with the analytical model and to study post-pull-in behavior. A stationary study was performed, up to the pull-in voltage. Beyond the pull-in voltage, a time dependent study was performed. The latter was based on the “Pull-in of an RF MEMS Switch” of the COMSOL Multiphysics${}^{®}$ library. The configuration of the model and the corresponding parameters used are shown in Figure 3.

### 2.3. Experimental Setup

**Figure 4:**
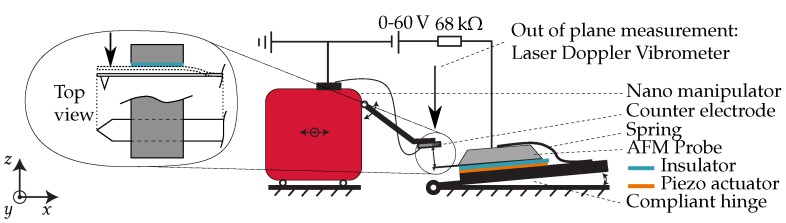
Experimental setup. A robotic nano-manipulator is used to position the electrode relative to a commercially available cantilever. The Atomic Force Microscopy (AFM) probe is actuated with a piezoelectric actuator and the frequency response is measured with a laser Doppler vibrometer for a varying electrode position along the length of the cantilever.

An experimental setup was developed to prove the change in stiffness of the cantilever. The setup is shown in Figure 4. A commercially available silicon probe was used for this experiment (BudgetSensors ContAl k = 0.2 N/m, E=169 GPa, ν=0.28). It was mounted on top of a stack of a prototyping PCB, a piezo actuator, an insulative 125 µm thick sheet of polydimethylsiloxane (PDMS) from Shielding Solutions. A small spring was used to apply a clamping force on this stack. A remotely controlled robotic nanomanipulator (miBot) from Imina Technologies was used to position the electrode relative to the cantilever. This nanomanipulator can move in-plane and has an arm that moves out-of-plane. This arm determined the gap between the electrode and the cantilever. The electrode was made by laser cutting of a 150 µm thick sheet of spring steel. It was covered by a 1.6 µm layer of photo resist (AZ9260), with a dielectric constant of 4.03 and dielectric strength of 694 V/µm. It was important that this layer had a high dielectric strength, such that the insulative properties remain intact when the cantilever was pulled-in. The electrode and cantilever were made parallel (confirmed from the optical image) by controlling the angle of the cantilever with a compliant hinge. In order to apply the bias voltage between the cantilever and electrode, electrical connections were made to connect the system to a voltage source of maximum 60 V. To protect the system in case of a short circuit, a current limiting resistor of 68 kΩ was added in series. The setup was placed in a Polytec MSA-400 laser Doppler vibrometer to measure the resonance frequency and modes of the cantilever. The piezo actuator under the cantilever was used to actuate the system, using a frequency sweep. The vibrometer measured the out of plane (*z*-axis in Figure 4) response of the cantilever for a varying electrode position. The fundamental resonance frequency was compared to the theoretical value. The built-in camera of the vibrometer was used to visualize the cantilever from the side, using a right angle mirror (N-BK7, Edmund optics). This enabled us to measure the effective cantilever length. More details of this experimental setup are given in the supplementary material.

## 3. Results

### 3.1. Modeling Results

**Figure 5:**
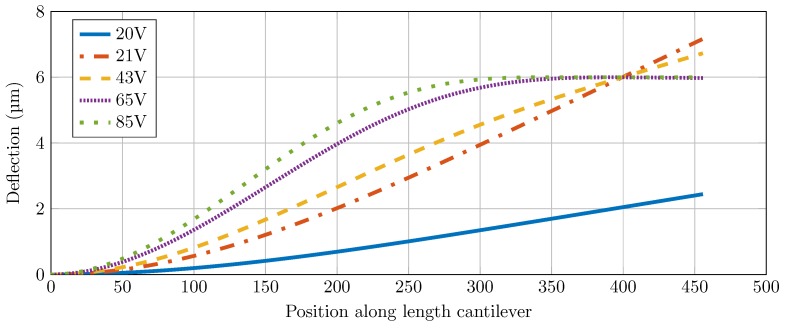
The shape of the cantilever is shown as a function of the position along the length of the cantilever. This is shown for a varying voltage.

**Figure 6:**
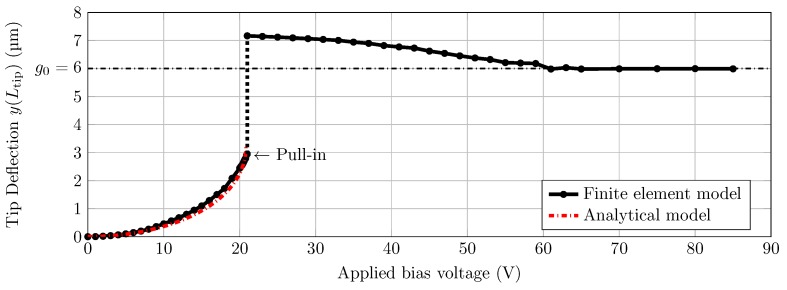
Modeling results. Tip deflection as a function of applied voltage. Up to pull-in the system is simulated as a stationary system. The analytical model shows similar results. After pull-in it is simulated as a time-depended study and the deflection at steady state is shown.

The shape of the cantilever for a varying voltage is shown in Figure 5. In Figure 6 the deflection at the tip in steady state is shown as a function of the bias voltage. The simulation results are compared with the analytical model. The pull-in voltage for the parameters used (Figure 3a) was found to be 21 V. When the voltage was further increased to 65 V, the system was in the clamped state. More details of the models and obtained results are given in the supplementary material.

### 3.2. Experimental Results

**Figure 7:**
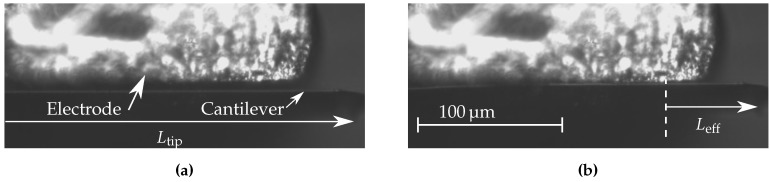
Side view of the electrode and the cantilever obtained with an optical microscope. (**a**) before stiffness adjustment; (**b**) after stiffness adjustment. The clamped side of the cantilever is located at the left (not shown). These positions correspond to the ones shown in Figure 2.

**Figure 8:**
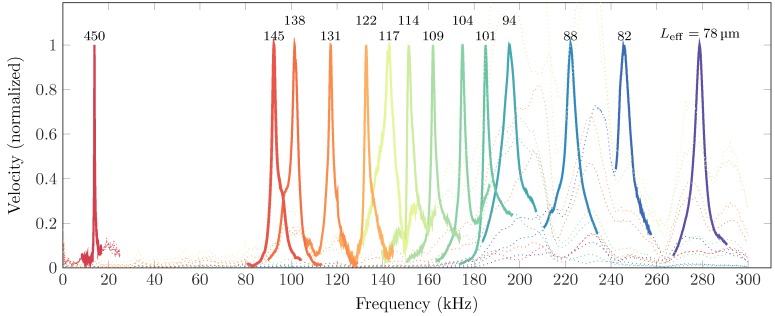
The fundamental resonance frequency obtained for different adjusted lengths of the cantilever. The electrode position is varied and the cantilever is pulled-in for these positions, reducing its effective length. The cantilever is actuated with a frequency sweep and the response is shown in this figure. The numbers above the peaks indicate the effective length of the cantilever (in µm). Only the resonance peaks of the cantilever are shown in a solid line, while the rest of the bandwidth is plotted as a dotted line for clarity. All the measurements were made at an applied voltage of 60 V. More data and a detailed discussion is given in the supplementary material.

**Figure 9:**
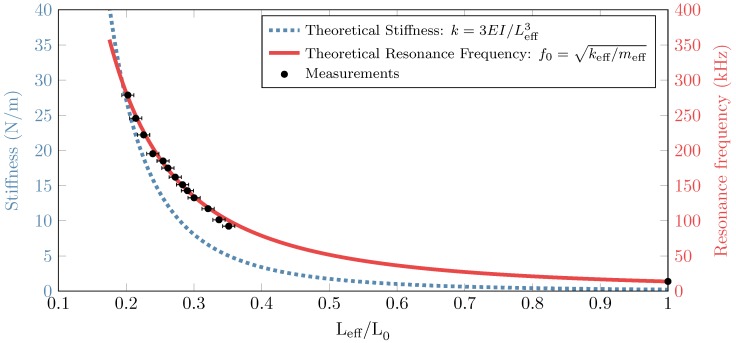
Theoretical stiffness and resonance frequency compared with the measurements as a function of the normalized effective length. The measurement points correspond with the peaks found in Figure 8. The error bars correspond to the measurement uncertainty on the effective length.

The position of the electrode relative to the cantilever ($L_{\mathrm{cel}}$) was varied by using the nano-manipulator. For each position the cantilever was pulled-in as shown in Figure 7. The frequency response was measured for each of these positions (Figure 8). The resonance peaks were clearly identified from the response obtained and are highlighted in solid. The dotted lines show the response outside of the resonance peaks, and comprises characteristics of the entire setup. This plot shows that the resonance frequency increases for decreasing effective length, as shown on top of the peaks. The measured resonance frequency that is shown in Figure 8 is compared with the theoretical resonance frequency in Figure 9. The corresponding theoretical stiffness is indicated as well, to show which value of stiffness corresponds to a certain value of resonance frequency. The effective length was measured (see Figure 7) with an optically calibrated microscope. The error bars indicate the uncertainty on the measurement of the effective length. The data points between $L_{\mathrm{eff}}=450$ to 145 µm are missing because the size of the electrode used did not allow to reach these values. However, the data fitting signifies that the concept should be valid in this region. More details and results are shown in the supplementary material.

## 4. Discussion

The goal of this work was to show that the stiffness of the cantilever could be adjusted *in-situ* by adjusting the effective length. We have adopted electrostatic pull-in method to prove this concept. Modeling of the concept gave a good initial insight into the behavior of the cantilever with electrostatic pull-in (Figure 5 and Figure 6). It also gave an initial estimate on the working distances and the applied voltage needed for our experiments. Initially, the situation is analogous to a parallel plate with uniform electric field between the plates. As the applied voltage increased, the free end of the cantilever starts bending creating a non-uniform gradient electric field between the cantilever and the electrode. The analytical equations from Equation (2) describe this situation. The results from these equations and FEM simulation match quite well proving their validity (Figure 6). However, these equations do not describe the tip deflection beyond pull-in voltage. To understand the cantilever behavior for applied voltage beyond the pull-in voltage, FEM simulations were used (see supplementary material for more details).

The high precision nano-manipulator was used to place the electrode very close to the cantilever. The electrode used on top of the cantilever was manipulated by observing the cantilever through a microscope having a top view of the cantilever. A right angle mirror was used to have a side view of the electrode-cantilever system to adjust the gap between them. In Figure 7 it is shown that the cantilever pulls in as expected in our simulated results. In Figure 8 the frequency response of the cantilever is shown for a varying effective length. It is shown that the resonance frequency of the cantilever shifts to higher values, when the effective length of the cantilever is reduced. The measurement point that are shown in this figure are used in Figure 9 to show the change in resonance as a function of the change in effective length. This is compared with the theoretical resonance frequency. The measurements match with the theoretical estimation quite well. The error bars on the measurements indicate the uncertainty on the optical measurement on the effective length. The resolution is limited, and the exact point of clamping is not exactly known, due to the curved shape of the electrode near the edge (Figure 7). It is assumed that this point is known within ±10 pixels, which is a conservative estimate. The uncertainty on the resonance frequency of the measurement is determined by the resolution of the laser Doppler vibrometer, which is 78.125 Hz for the bandwidth that was used for the measurements. This is negligible compared to the measured frequencies. The missing data in the plot shown in Figure 9 for $0.4<L_{\mathrm{eff}}/L_{0}<1$ is due to the big electrode width compared to the cantilever length. The width is limited by the conventional fabrication method used. The data can be obtained if the electrode width is reduced, which can be achieved by microfabrication methods. During the measurements it was observed that when the electrode was in close proximity (5–10 µm), but no voltage was applied, measurement of the first resonance mode showed heavy damping. This could be due to squeeze-film damping. The air trapped between the cantilever and electrode in the narrow gap increases the viscous forces on the cantilever [18] (see supplementary material). For accurate quantitative data the damping in the system must be properly characterized, which will be challenging as it is changing for every cantilever configuration and we leave it for future analysis.

With the proposed technique, when operated in real AFM, as the cantilever clamps to the electrode, the effective distance from the cantilever to the photo-detector would change. Hence the optical lever sensitivity will be slightly reduced from the unclamped situation to the clamped situation. This could also lead to change in the laser position on the detector, and may even go beyond the detector zone. However, the cantilever shifts by the same distance for all the clamped situations. Therefore, for all the clamped situations, the optical sensitivity should remain same. Defining two sensitivities would suffice for this method: One for the unclamped condition and the other for the clamped condition. Once the cantilever is clamped, thermal resonance spectra can be measured, and by standard methods like Sader method, the stiffness of the cantilever can be obtained. With this information, the force sensitivity for each stiffness value can be calculated. Another aspect to note is that as the cantilever goes away from the surface, the effective tip height is compromised. One of the solutions could be to slightly change the angle of mounting to minimize the compromised range. This can be integrated in the chip design itself or use an adapter on the mounting stage of the AFM setup.

Due to continuous bending of the cantilever, fatigue in the cantilever could be a concerning factor. There are numerous reports showing that fatigue in silicon has resulted in its failure [19,20,21]. This can lead to permanent deformation or complete damage to the cantilever. Maintaining the life cycle of the cantilever for its stiffness properties should give information on the fatigue. Any internal structural damage leading to fatigue should reflect in the resonance frequency of the cantilever concluding the “health” of the cantilever. The repeated use of the method might also lead to some damage to the metallic coating used for laser deflection measurements.

Stiction of the cantilever to the electrode was not observed most of the time. However, in some cases the cantilever was not restored to its unadjusted state after the voltage was released. In order to prevent this, precautions like decreasing contact area by surface roughening, incorporating dimples or chemically modifying the surface with an anti-stiction coating are recommended [22].

For operation in liquids, one of the potential problems that can be foreseen is the principle of electrostatics itself. For biological samples, which are usually kept in buffer solutions, the conductivity of the buffer might influence the intended function of clamping the cantilever. However, the principle should work in non-conducting solutions. One of the potential solution to work in conducting solutions could be to use the method proposed by Ziegler *et al.* by encasing the cantilever inside a hollow structure [23]. With a hollow casing around the cantilever and making the cantilever and the casing completely hydrophobic, the entire cantilever can be immersed inside biological liquids. The stiffness tuning mechanism should be integrated inside the channel. This could be quite challenging though.

## 5. Conclusions

The concept of a stiffness adjustment in a commercial AFM probe was presented. The change in stiffness was achieved by a change in effective length by means of electrostatic pull-in. Analytical model till pull-in voltage and FEM simulation of the entire working range was presented. An experimental setup was built to prove the concept. By reducing the unadjusted length of a 450 µm long cantilever to 78 µm long in many intermediate steps, the stiffness was shown to change between 0.2 N/m and 27 N/m covering two orders of magnitude in a single cantilever. As the present setup was not compatible with any commercial AFM setup, no real AFM experiments were performed. However, the concept of stiffness adjustment with commercial AFM cantilevers has been proven. The next step would be to design and microfabricate a prototype that integrates the cantilever and a movable electrode in a single device. The ultimate goal of *in situ* stiffness adjusted cantilever will be to cover the entire stiffness range used in various modes of AFM operation (Figure 1) in a single cantilever. Such a device can make multi-parameter AFM measurements easier, faster and more versatile.

## Supplementary Files

Supplementary File 1
