# Supplementary material for: In situ Stiffness Adjustment of AFM Probes by Two Orders of Magnitude
Source: Sensors (Basel). 2016 Apr 12;16(4):523. doi: 10.3390/s16040523 (PMC4851037; doi:10.3390/s16040523)
Supplement: Supplementary File 1 [file sensors-16-00523-s001.pdf]

Supplementary Materials: *In Situ* Stiffness Adjustment of AFM Probes by Two Orders of Magnitude

Marcel Lambertus Cornelis de Laat, Héctor Hugo Pérez Garza and Murali Krishna Ghatkesar

1. Supplementary Material: Characterization of Electrostatic Pull-in Behavior of AFM Probes

In order to get a good understanding of the system a study has been performed on the voltage-deflection behavior of a cantilever under an electrostatic load. Experiments were performed on a commercially available cantilever. These measurements were compared with models. In Section 1.1 the used probes are characterized.

Two models were developed to study the system. The first model is an analytical model, that is based on previous work in literature [1], which is described in Section 1.2. The second model is a COMSOL model which is shown in Appendix B.1. These models are compared with measurements that were performed on a commercially available AFM cantilever, which is elaborated in Section 2.3.

In order to facilitate the modeling, the shape of the cantilever is simplified according to Figure S1. The shape of the cantilever near the tip is triangularly shaped from top view. This triangle starts at L_{side} and ends at L_{tot}. The tip is located at L_{tip}, which is approximately at the center of this triangle. For the models it is assumed that the cantilever has a constant cross section and has length L_{tip}.

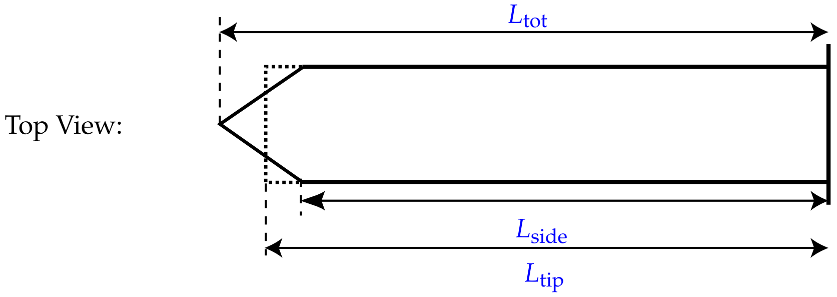

Figure S1. Approximation of cantilever geometry for modeling. The solid line is the actual geometry near the tip, while the dashed line shows the approximation used for modeling.

1.1. Cantilever Characterization

In order to make a fair comparison between models and measurement, it is important to know the dimensions and properties of the cantilever as accurate as possible. These properties can then be used as input for the model. The dimensions which are in the specifications of the product have a large tolerance as shown in Table S1. The aluminum reflex coating is neglected. The most important parameters are the geometry of the cantilever (length and thickness) and material properties, because these determine the stiffness of the cantilever. In Section 1.1.1 the material properties of the cantilever are presented. In Section 1.1.2 the method for measuring the length and with of the cantilever is shown. The measurement of the thickness is explained in Section 1.1.3.

Table S1. Specifications of the cantilever from the supplier.

Property	Value	Range
Resonance frequency	13 kHz	±4 kHz
Force constant	0.2 N·m^{-1}	0.07 to 0.4 N·m^{-1}
Length	450 µm	±10 µm
Mean Width	50 µm	±50 µm
Thickness	2 µm	±1 µm
Tip Height	17 µm	±2 µm
Tip Set Back	15 µm	±5 µm
Tip Radius	<10 nm	
Reflex coating	Aluminium relfex coating 30 nm thick	
Half Cone Angle	20°–25° along cantilever axis<br>25°–30° from side<br>10° at the apex	

1.1.1. Material Properties

The Young's modulus in silicon is anisotropic, in each crystallographic direction the value is different [2]. After inquiry at the manufacturer of the used probes, the cantilevers point in the $\langle 110 \rangle$ direction. This corresponds to a Young's modulus of 169 GPa. For wide, thin beams ($w \geq 5t$) an effective Young's modulus $\hat{E}$ is used. This is defined as $\hat{E} = E \setminus (1 - \nu^2)$, with ν the Poisson's ratio. Also the Poisson's ratio is not constant in all directions. For the out of plane bending of the cantilever: $\nu = 0.28$. The density of silicon is 2320 kg·m^{-3}.

1.1.2. Length and Width

The length and width of the cantilevers are measured with the Scanning Electron Microscrope (SEM). Since the cantilever has a trapezoidal cross section, this measurement is done both on the top- and bottom-side. The definition of these dimensions are shown in Figure S2. The results are summarized in Table S2. A typical set of images, used to determine the dimensions are shown in Appendix A. These dimensions are assumed to be measured with ±0.5 µm accuracy.

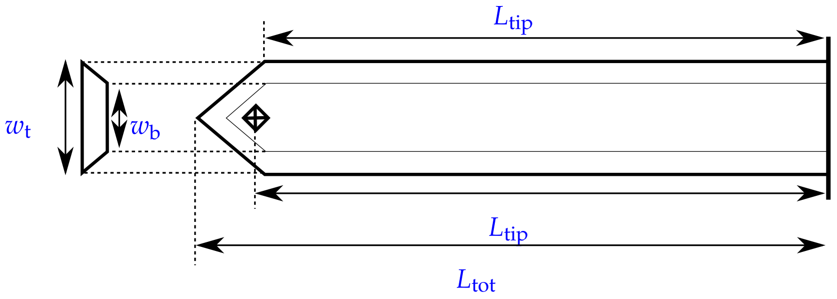

Figure S2. The definition of the dimensions that were measured with the SEM.

1.1.3. Thickness

In contrast to the length and the width, the thickness is not measurable with the SEM. Because of the trapezoid shape of the cross section of the cantilever, it is not possible to have the entire thickness in focus due to the limited depth of focus. The thickness can be calculated by measuring the resonance frequency, and solving the following equation for the thickness which is the only unknown parameter:

$$f_0 = \frac{1}{2\pi}\sqrt{\frac{k}{m_{\text{eff}}}} \tag{1}$$

$$= \frac{1}{2\pi}\sqrt{\frac{3EI(t)}{m_{\text{eff}}(t)}} \tag{2}$$

with:

$$I = \frac{t_c{}^3}{36}\frac{w_t{}^2 + 4w_t w_b + w_b{}^2}{w_t + w_b} \tag{3}$$

$$m_{\text{eff}} = m_{\text{tip}} + \frac{33}{140}m_{\text{cant}} \tag{4}$$

$$= m_{\text{tip}} + \frac{33}{140}(\rho_s L_{\text{tip}} t_c \frac{w_t + w_b}{2}) \tag{5}$$

The resonance frequency can be determined very accurate with a laser Doppler vibrometer. The probe is placed on a piezoelectric actuator. A frequency sweep is applied to the actuator and the response of the cantilever is measured. The resolution of the vibrometer is 3.12 Hz.

The exact shape of the cantilever needs to be approximated, because the complex geometry near the end of the cantilever cannot be taken into account by this model. The simplified model is shown in Figure S3. The volume which is indicated in gray is assumed to be a concentrated mass at L_{tip}. This consists of the tip itself and the triangular end facet of the cantilever. This part is approximated by a triangle with a base width of $\frac{w_t+w_b}{2}$. The volume of the tip is hard to estimate, because the shape is complex. It is approximated by a pyramid shape, with a base of 6 μm and a height of 15 μm, which is estimated with the SEM. The volume of a pyramid is: $V = \frac{l \times w \times h}{3}$. It is assumed that this volume is determined within ±20 % accuracy.

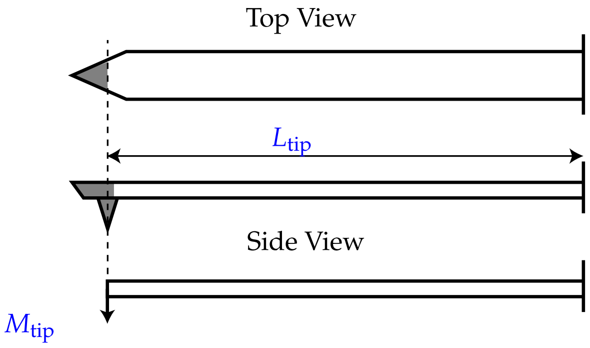

Figure S3. The model of the cantilever to calculate the thickness as a function of its resonance frequency. The gray parts are considered to be a concentrated mass at the end of the cantilever. The length is assumed to be equal to the length upto the tip.

1.1.4. Conclusions

The length and with of the cantilever were measured by using a SEM. The thickness of the cantilever is determined by measuring the resonance frequency with a laser Doppler vibrometer, and solving Equation (2) for the thickness. The measurements with the SEM are assumed to be accurate within ±0.5 μm. The measurement of the resonance frequency is assumed to be accurate within ±3 Hz. The volume, which is assumed to be a concentrated mass at L_{tip} is assumed to be accurate within ±20%. The measured dimensions, resonance frequencies and resulting thickness and confidence interval are shown in Table S2.

Table S2. Dimensions and resonance frequency of the used cantilevers. This data is acquired by measurement with a Scanning electron microscope and laser doppler vibrometer. The accuracy of the dimensions is assumed to be ±0.5 µm, for the resonance frequency ±3.12 Hz.

Cantilever	L_{side} (µm)	L_{tip} (µm)	L_{tip} (µm)	w_t (µm)	w_b (µm)	f_0 (Hz)	t_c (µm)
C1	438.8	453.0	471.0	57.8	46.6	13,793.75	2.045 ± 0.029
C3	436.0	446.0	476.5	57.7	46.2	13,409.38	1.981 ± 0.037
C5	437.0	450.6	473.4	57.7	46.8	13,481.25	1.999 ± 0.032
C7	443.4	455.7	476.9	57.8	46.7	13,265.63	2.004 ± 0.030

1.2. Analytical Model

Pull-in behavior of micro cantilevers is a well researched topic. Several approaches were presented like simplified closed-form analytical models [3–6], numerical method of solving differential equations [7] and a semi-analytical approach [1]. These (semi-) analytical models provide an advantage over finite element models and numerical methods in terms of speed of calculation, but require more simplifications that might yield less accurate results. The model developed by Do *et al.* [1] is chosen for the modeling of the system; it includes the possibility to include a partially covering electrode, incorporates fringe fields and a non-uniform electrostatic load. First, the original model will be presented in Section 1.2.1. In Section 1.2.2 a modification of the model is presented to adapt it to the configuration of the experiments.

1.2.1. The Original Model

The schematic view of the model is shown in Figure S4a, and the cross section of the cantilever in Figure S4b. It is assumed that the cantilever has a constant cross section. The cantilever of length L_{tip} is fixed on the left side. An electrode of length L_{el} is covering a part of the cantilever. The deflection at each point along the cantilever is described by $y(x)$, and the deflection at the tip as $y(L_{tip})$. The gap g_0 is defined as the distance between electrode and cantilever in undeflected state. The parameter α is the ratio between the length of the electrode and the length of the cantilever.

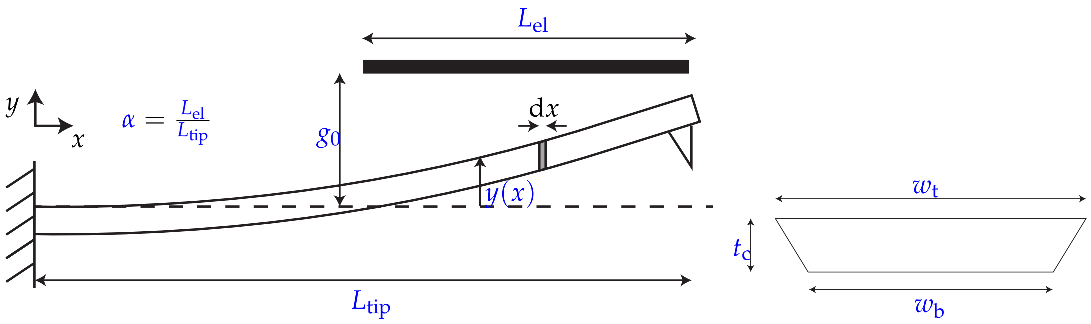

(a) Schematic side view with electrode covering a part of the cantilever. The analytical model is based on this schematic.

(b) Cross section of the cantilever.

Figure S4. Schematic drawing of the model, inspired by [1].

The electrostatic force on a small element of the cantilever of length dx is defined as:

$$q_{elec}(x) = \frac{\epsilon_{air} w_{eff} {V_{DC}}^2}{2(g_0 - y(x))^2} \mathrm{d}x \quad (6)$$

where V_{DC} is the applied potential between electrode and cantilever and w_{eff} is the effective width of the cantilever, compensated for fringe field effects [8]:

$$w_{\mathrm{eff}} = w_{\mathrm{t}}(1 + 0.65\frac{(1 - y(L_{\mathrm{tip}})/g_0)g_0}{w_{\mathrm{t}}}) \tag{7}$$

The deflection at the tip of a cantilever due to a distributed force is given by [9]:

$$\mathrm{d}y(L_{\mathrm{tip}}) = \frac{q_{\mathrm{elec}}(x)x^2(3L - x)}{6\hat{E}I} \tag{8}$$

where $\hat{E}$ is the effective Young's modulus ($\hat{E} = E \setminus (1 - \nu^2)$ with ν Poisson's ratio, for wide beams ($w_{\mathrm{c}} \geq 5t_{\mathrm{c}}$)) and I the second moment of area. The paper assumes a rectangular shaped cantilever, while in the present work a trapezoidal cantilever is used. To incorporate this, the following expression will be used [7]:

$$I = \frac{{t_{\mathrm{c}}}^3}{36}\frac{{w_{\mathrm{t}}}^2 + 4w_{\mathrm{t}}w_{\mathrm{b}} + {w_{\mathrm{b}}}^2}{w_{\mathrm{t}} + w_{\mathrm{b}}} \tag{9}$$

where t_{c} is the thickness, w_{t} and w_{b} the width at the top and bottom respectively.

The deflection at the end of the cantilever is found by substituting Equation (6) in Equation (8) and integrating over the region where the electrode and cantilever overlap:

$$y(L_{\mathrm{tip}}) = \frac{\epsilon_{\mathrm{air}}w_{\mathrm{eff}}{V_{\mathrm{DC}}}^2}{12\hat{E}I}\int_{L_{\mathrm{tip}}-\alpha L_{\mathrm{tip}}}^{L_{\mathrm{tip}}} \frac{x^2(3L_{\mathrm{tip}} - x)}{(g_0 - y(x))^2}\mathrm{d}x \tag{10}$$

The total force is found in a similar way, by integrating the distributed load Equation (6) over the overlapping section:

$$\vec{F}_{\mathrm{e}} = \int_{L_{\mathrm{el}}} q_{\mathrm{elec}}(x) = \frac{\epsilon_{\mathrm{air}}w_{\mathrm{eff}}{V_{\mathrm{DC}}}^2}{2}\int_{L_{\mathrm{tip}}-\alpha L_{\mathrm{tip}}}^{L_{\mathrm{tip}}} \frac{1}{(g_0 - y(x))^2}\mathrm{d}x \tag{11}$$

So the effective non-linear stiffness of the entire system can be expressed as:

$$K_{\mathrm{eff}}(y(L_{\mathrm{tip}})) = \frac{\vec{F}_{\mathrm{e}}}{y(L_{\mathrm{tip}})} \tag{12}$$

$$= 6\hat{E}I\frac{\int_{L_{\mathrm{tip}}-\alpha L_{\mathrm{tip}}}^{L_{\mathrm{tip}}} 1/(g_0 - y(x))^2\mathrm{d}x}{\int_{L_{\mathrm{tip}}-\alpha L_{\mathrm{tip}}}^{L_{\mathrm{tip}}} x^2(3L_{\mathrm{tip}} - x)/(g_0 - y(x))^2\mathrm{d}x} \tag{13}$$

Pull-in will occur when the electrostatic force exceeds the mechanical restoring force of the cantilever. This point of instability can be found at:

$$\vec{F}_{\mathrm{e}} = K_{\mathrm{eff}}y(L_{\mathrm{tip}}) \tag{14}$$

Solving this equation for the applied voltage yields:

$$V_{\mathrm{DC}} = \sqrt{\frac{12\hat{E}Iy(L_{\mathrm{tip}})}{\epsilon_{\mathrm{air}} w_{\mathrm{eff}} \int_{L_{\mathrm{tip}}-\alpha L_{\mathrm{tip}}}^{L_{\mathrm{tip}}} \frac{x^2(3L-x)}{(g_0-y(x))^2}\mathrm{d}x}} \tag{15}$$

In order to solve this equation, the shape of the cantilever should be known. This is approximated using a FEM extracted shape function:

$$y(x) = \left(2.56 - \frac{16.127}{4(x/L_{\mathrm{tip}} + 0.00185)^2 + 6.2786}\right) y(L_{\mathrm{tip}}) \tag{16}$$

This shape is validated with COMSOl results in Section 2.2. A numerical method was presented to find the closed form of the integral. For this thesis the closed-form is not used; Matlab® is used to compute the complicated integral for the FEM extracted shape function.

1.2.2. Extending the Model for the Current System

The model as presented in the previous section should be extended such that it can handle a partially covering electrode and an insulative layer, as shown in Figure S5a. The original model assumes that the end of the electrode and cantilever are aligned. But in the experimental setup this is not the case. In the new model it is assumed that the last part of the cantilever is protruding. The part of the cantilever up to the edge of the electrode is defined as $L_{\text{c-el}}$. Fringe field effects at this edge are neglected. The equation for the distributed electrostatic load (Equation (6)) should include the influence of the insulating layer. The distributed electrostatic force $q_{\mathrm{elec}}(x)$ is depending on the permittivity of the insulator ϵ_{ins} and layer thickness d_{ins}. (Note that the permittivity in a certain medium is defined as the product of the absolute and relative permittivity. For air the relative permittivity is equal to one, so $\epsilon_{\mathrm{air}} = \epsilon_0$. The permittivity in the insulator is: $\epsilon_{\mathrm{ins}} = \epsilon_{\mathrm{r}}\epsilon_0$.) If we consider the situation of Figure S5a, a small section of length $\mathrm{d}x$ can be shown as Figure S5b. The displacement vector $\vec{D}$ is equal in both the insulator and the air gap, and the total voltage V_{DC} is the sum of the voltage drop over the insulator and air gap ($g = g_0 - y(x)$):

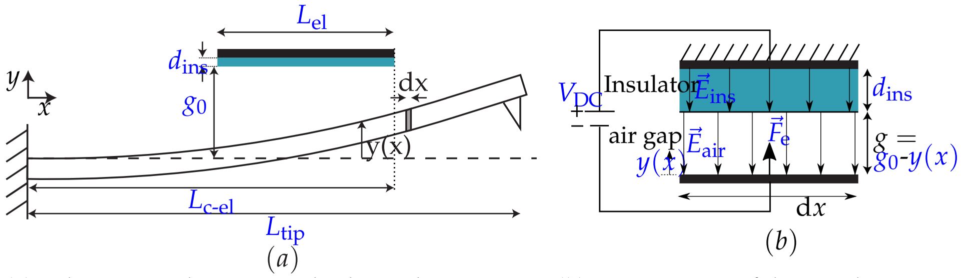

(a) Schematic side view with electrode covering a part of the cantilever. The electrode is covered with an insulative layer.

(b) Cross section of the cantilever.

Figure S5. Modified schematic of the model such that it includes a dielectric layer and a protruding part of the cantilever.

$$V_{\mathrm{DC}} = V_{\mathrm{ins}} + V_{\mathrm{air}} \tag{17}$$

$$= \vec{E}_{\mathrm{ins}} d_{\mathrm{ins}} + \vec{E}_{\mathrm{air}} g_0 \tag{18}$$

$$= \frac{\vec{D}}{\epsilon_{\mathrm{ins}}} d_{\mathrm{ins}} + \frac{\vec{D}}{\epsilon_{\mathrm{air}}} g = \vec{D}\left(\frac{\epsilon_{\mathrm{air}} d_{\mathrm{ins}} + \epsilon_{\mathrm{ins}} g}{\epsilon_{\mathrm{ins}}\epsilon_{\mathrm{air}}}\right) \tag{19}$$

So the displacement vector $\vec{D}$ is be expressed as:

$$\vec{D} = \frac{V_{DC}\epsilon_{ins}\epsilon_{air}}{\epsilon_{air}d_{ins} + \epsilon_{ins}g} \quad (20)$$

Now the electric field in the air gap can be derived from the displacement vector $\vec{D}$ and the force on the plate can be calculated:

$$\vec{E}_{air} = \frac{V_{DC}\epsilon_{ins}}{\epsilon_{air}d_{ins} + \epsilon_{ins}g_0} \quad (21)$$

$$\vec{F}_e = \frac{\epsilon_{air}\vec{E}_{air}{}^2 A_{cap}}{2} = \frac{\epsilon_{air}\epsilon_{ins}{}^2 V_{DC}{}^2 A_{cap}}{2(\epsilon_{air}d_{ins} + \epsilon_{ins}g_0)^2} \quad (22)$$

If this electric force is considered for a small slice width w_t and length $\mathrm{d}x$, and gap $g(x) = g_0 - y(x)$, the distributed electrostatic force $q_{elec}(x)$ is expressed as:

$$q_{elec}(x) = \frac{\epsilon_{air}\epsilon_{ins}{}^2 V_{DC}{}^2 w_t \,\mathrm{d}x}{2(\epsilon_{air}d_{ins} + \epsilon_{ins}(g_0 - y(x)))^2} \quad (23)$$

If we substitute Equation (23) in Equation (8) and perform the same steps as were described in Equations (10)–(14), the required voltage for equilibrium for a given deflection is:

$$V_{DC} = \sqrt{\frac{12\hat{E}Iy(L_{c\text{-}el})}{\epsilon_{air}\epsilon_{ins}{}^2 w_{eff} \int_{L_{c\text{-}el}-\alpha L_{c\text{-}el}}^{L_{c\text{-}el}} \frac{x^2(3L_{c\text{-}el}-x)}{(\epsilon_{air}d_{ins}+\epsilon_{ins}(g_0-y(x)))^2}\mathrm{d}x}} \quad (24)$$

The deflection at the tip $y(L_{tip})$ is found by linearly extrapolating the deflection at $y(L_{c\text{-}el})$. It is plotted in the Figure S6.

$$y(L_{tip}) = y(L_{c\text{-}el}) + \left.\frac{\mathrm{d}y(x)}{\mathrm{d}x}\right|_{L_{c\text{-}el}} (L_{tip} - L_{c\text{-}el}) \quad (25)$$

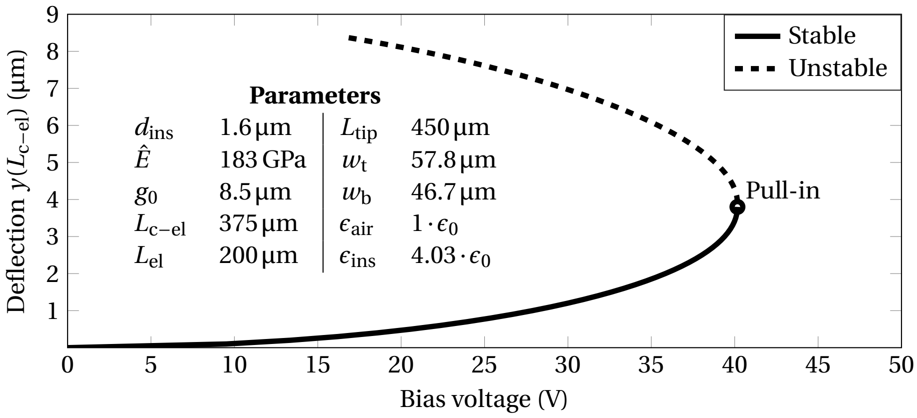

Figure S6. The result of Equation (25). The parameters which are used are shown in the figure. The solid line indicates the stable state of the system, while the dashed line shows the unstable solution of the system. At pull-in, the electrostatic forces exceed the mechanical restoring forces.

2. FEM Model in COMSOL

In this appendix the COMSOL model will be discussed in more detail. The physics, geometry, study and post processing will be discussed. The model is based on the "electrostatic actuated cantilever" that can be found in the COMSOL model library.

2.1. Static Model

Physics The physics that were used is "electromechanics". This is needed to simulate the mechanical behavior of the cantilever and the electrostatic force caused by the applied potential.

Geometry The first step is making a plane geometry of the cross section of the cantilever and the surrounding air. This is shown in Figure S7. The cantilever itself is made with a polygon, while the air is made with a rectangle. Another rectangle is used to model the insulative layer. An extra line, splitting the cantilever in two, is added to make sure there is a mesh boundary at this point. This can be used to plot the cross-section. Next, the planar geometry is extruded, which is shown in Figure S8. This is done in two steps; region without overlap with electrode and a region with overlap up to L_{side}. An extra block is added for the air, surrounding this part of the cantilever. This block is 10 µm longer than the length of the cantilever. The insulative layer is made with two hexahedrons.

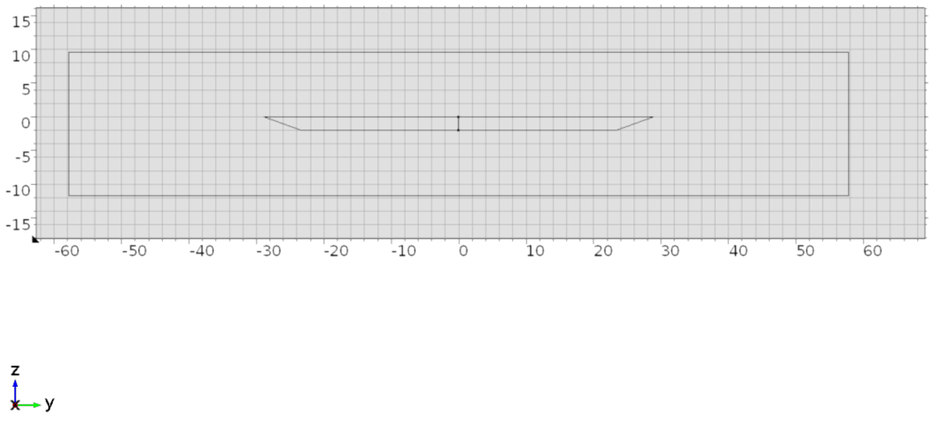

Figure S7. The cross section of the cantilever and surrounding air modeled in COMSOL.

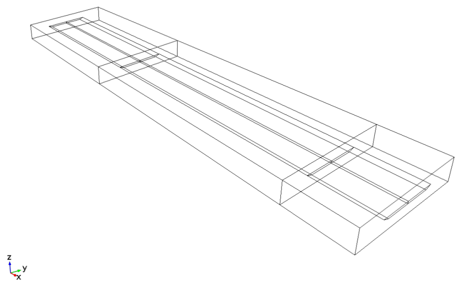

(a) The extrusion of the planar geometry of Figure S7 in different stages.

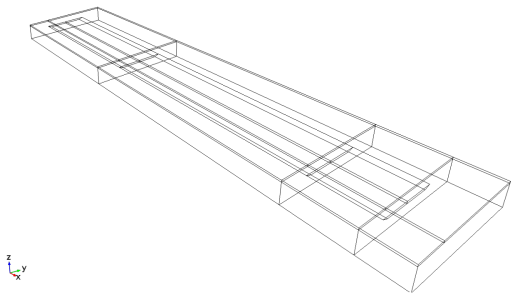

(b) The complete geometry.

Figure S8. The 3D design of the cantilever in COMSOL.

Materials and physics The material of the cantilever is silicon, the insulative layer is a material with relative permittivity of 4.03 and the remaining domains are made of air. The default material properties are used. The most important properties are the relative permittivity of air of 1, Young's

modulus of silicon 169 GPa and Poisson's ratio of silicon 0.28, which is equal to the values used for the analytical model.

The base of the cantilever is chosen to be a fixed constraint and the static potential is applied to the entire top surface of the cantilever.

Mesh Next, the mesh is generated. Because a large part of the geometry has a constant cross section, a planar, quad mesh is applied to the plane at the base as shown in Figure S9a, that is swept through the geometry as shown in Figure S9b. A mesh convergence study has been performed, by comparing course and fine meshes. A finer mesh should give more accurate results, while a course mesh provides fast calculation. The results will converge for an increasing amount of elements, but more elements do not necessarily result in a more accurate model; the location of the elements is an important fact to consider. The mesh of the planar geometry and the swept are varied, with the predefined settings in COMSOL ("normal", "fine", "extra fine" *etc.*). In order to make a comparison between the different meshes, the tip displacement is used as the parameter of comparison. And all the meshes are solved in a static case, where a potential of 25 V is applied. The results of the convergence study are summarized in Table S3, where the results are organized according to the number of elements.

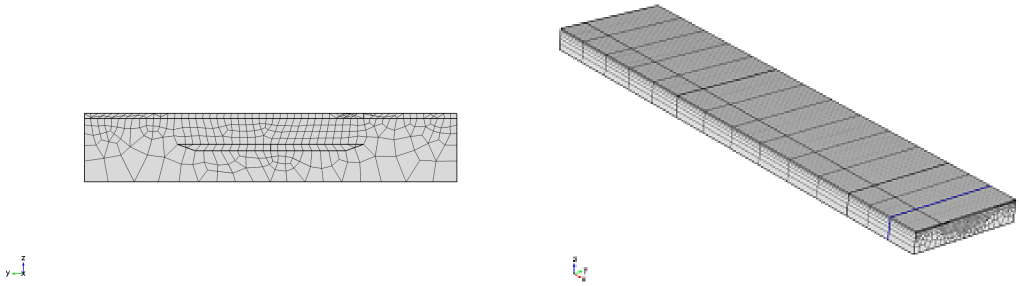

(a) The extrusion of the planar geometry of Figure S7 in different stages. **(b)** The complete geometry.

Figure S9. The 3D design of the cantilever in COMSOL.

Table S3. Results of the mesh convergence study. Where the refinement of the parts of the mesh (planar, swept and tetrahedral) are used as variables for the number of degrees of freedom (DOF) and maximum displacement that was found.

Number	Planar Mesh	Swept	DOF	Tip Deflection (μm)
1	extra fine	fine	173,155	1.824
2	fine	extra fine	166,131	1.875
3	finer	fine	117,059	1.710
4	fine	fine	76,507	1.8805
5	normal	fine	41,895	1.647

2.2. Comparison of the Models

In order to check whether the analytical- and COMSOL are correct, these models are compared. If the results are similar, it is likely that they are correct. The analytical model is based on a shape function (Equation (16)), which was extracted from FEM results by Do *et al.* [1]. In Figure S10 the shape of the analytical model and COMSOL model are compared for three different values of tip deflection ($y(L_{tip})$). The shapes are very similar. The difference between the shape functions is shown in Figure S11. The difference between the shapes increases for an increasing tip deflection. The maximum error for these shape functions is 15%, which is located at 120 μm. In the experiments, the electrode is covering the cantilever between ∼141 μm and ∼370 μm. The shape function only has an influence on the voltage-deflection behavior between this range. The error between this range is smaller than the

maximum error. It is concluded that the used function is an appropriate approximation for the shape of the cantilever.

In Figure S12a the deflection at the tip of the cantilever $y(L_{\mathrm{tip}})$ is shown as a function of the voltage. The two models give a very similar result. The COMSOL model has a slightly higher deflection for any given voltage. The analytical model only considers fringe field effects along the length of the cantilever, while in the COMSOL model the fringe fields along the width of the cantilever are also taken into account as shown in Figure S12b–d. These fringe fields add an extra electrostatic force to the system, yielding a slightly higher force for the same voltage for the COMSOL model.

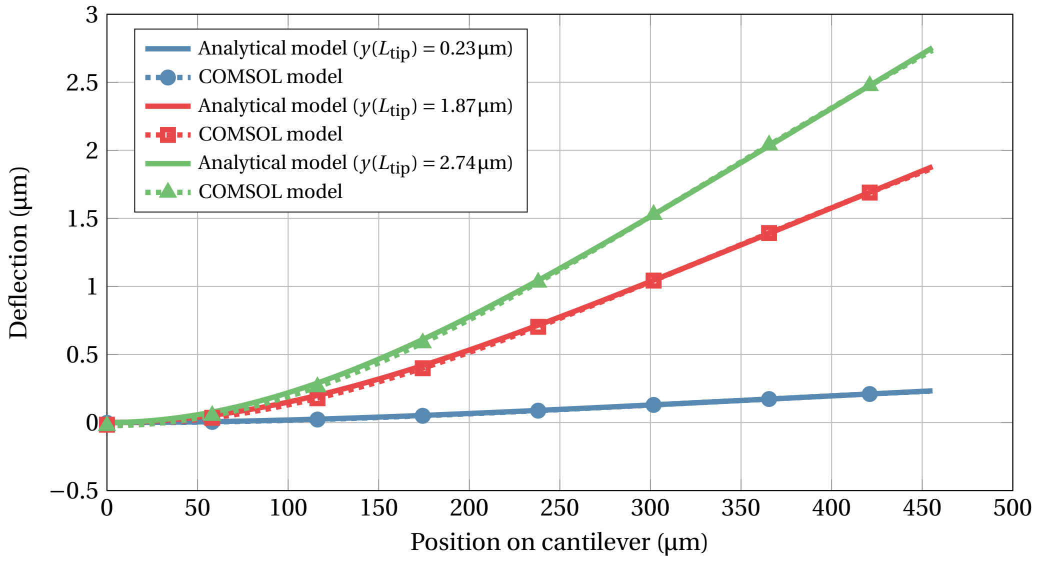

Figure S10. Comparison between the analytical model and COMSOL model on the shape of the cantilever for a varying deflection.

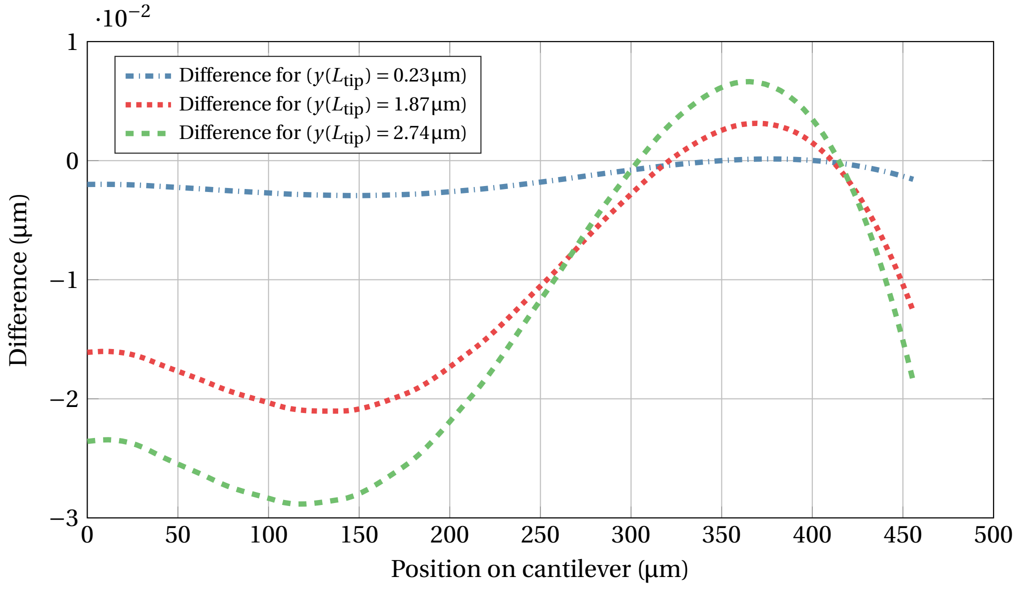

Figure S11. The difference between the analytical model and COMSOL model. These plots represent the difference between the corresponding lines of Figure S10. The difference, expressed as a percentage is constant for all three.

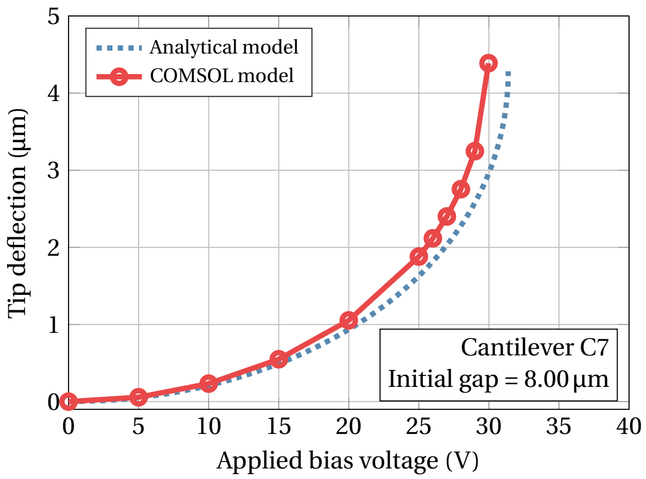

(a) The voltage-deflection behavior of a cantilever. The deflection at the tip is plotted versus the applied voltage.

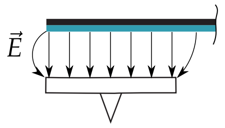

(b) Front view of analytical and COMSOL model. Both have fringe field.

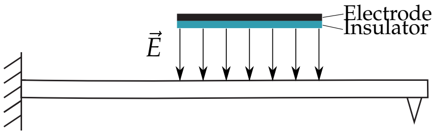

(c) Side view of analytical model.

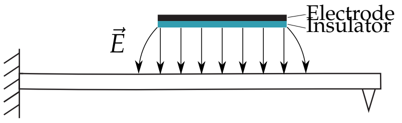

(d) Side view COMSOL model.

Figure S12. The assumptions on the fringe fields for the COMSOL- and analytical model are shown in Figure S12b–d. The analytical model assumes that there are no fringe fields near the edges of the electrode along the width of the cantilever. This is possibly the cause of the difference between the voltage-deflection behavior which is shown in Figure S12a.

2.3. Experimental Validation of the Models

In order to validate the models that were developed in Sections 1.2–2.2 an experiment was conducted. The materials and methods for this experiment are shown in Section 2.3.1. The results are shown inSection 2.3.2 and will be discussed in Section 2.3.3. The conclusions are found in Section 2.3.4.

2.3.1. Materials and Methods

In this section, we describe the following three topics: components used for the experimental setup, components integration and the measurement procedure and data processing.

AFM Probes

Commercially available cantilevers were used for the experiment. In order to reduce the required voltage for snap in cantilevers with a low stiffness and large top surface area were selected. The low stiffness reduces the required electrostatic force for pull-in. The large top surface area results in a relatively large electrostatic force. The used cantilevers are ContAl probes, manufactured by BudgetSensors. More details are found in Section 1.1.

Electrodes

The electrodes used for this experiments are made out of 150 µm thick spring steel and are machined using a LionMetal laser cutter, which was available at the PBM work shop at the faculty of Mechanical Engineering at Delft University of Technology. This laser cutter is not intended for micro meter accuracy, but by tuning the settings of the machine it was possible to manufacture sufficiently small features. The design of the electrodes is shown in Figure S13a. This design was made in Solid Works, and exported to a "*.dxf*"-file that can be read by the laser cutter. The strips of spring steel were secured on a 5 mm thick steel plate with tape and was placed in the machine as shown in Figure S13b. The resulting strip of spring steel is shown in Figure S13c. The resulting beams had a large variability of quality. Some of the beams broke during cutting, due to the strong air flow near the nozzle. Others were much wider or thinner than average. This is because the machine was operating

at the edge of its capabilities. The beams were examined in the Scanning Electron Microscope (SEM) as shown in Figure S14a. On the side of the nozzle there is a clean edge and surface. But on the other side, solidified metal forms a rough edge. The clean side is usable as electrode for the experiments.

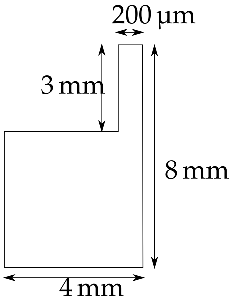

(a) Design of the electrode for the laser cutter.

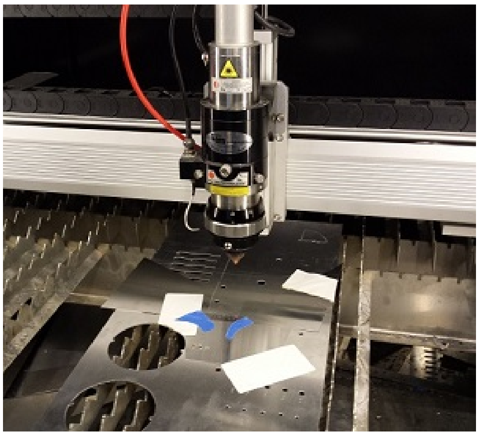

(b) The strip of spring steel secured on a plate of steel in the laser cutter chamber.

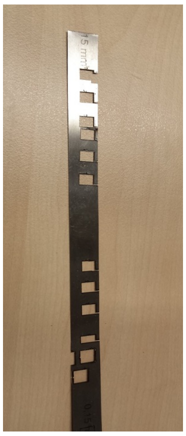

(c) Result of the strip of spring steel after laser cutting.

Figure S13. Manufacturing of the electrodes from spring steel with a laser cutter.

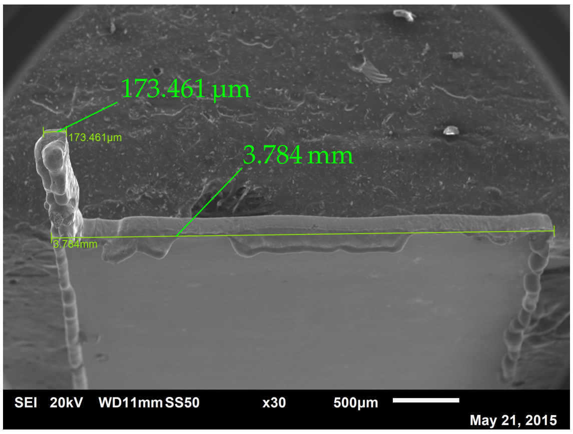

(a) SEM micrograph of the laser cut stainless steel part. The rough edges are solidified droplets of metal. These are formed on the opposite side of the nozzle of the cutter.

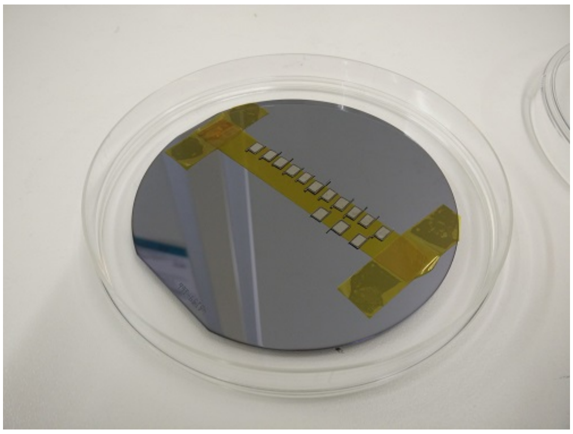

(b) Electrodes secured to a wafer with Kapton tape, before being processed in the spray coater.

Figure S14. The result of the electrode after laser cutting (**a**); The electrodes on a wafer before spray coating photo resist (**b**).

In order to be able to focus both the electrode and the cantilever in the optical microscope during the experiments, the end surface of the electrode should be as straight and smooth as possible, such that it remains within the focal depth of the microscope. In order to achieve this, the end of the electrode were cut with sharp pliers, and smoothed with sand paper. Afterwards they were cleaned with ultra sound in DI water (6 min) and iso propanol alcohol (IPA) (6 min).

The insulative coating was applied at the clean room facilities of EKL at Delft University of Technology. Photoresist was chosen as insulator, because it is cheap, easily applicable and has a high dielectric strength. The breakdown voltage is specified as 694 V·µm^{-1}. But in practice this might be significantly lower due to bad film quality. It was applied with an EVG 101 spray coater, using

a 4 layer 1000 mbar 2 mL recipe. The electrodes were mounted on a wafer with Kapton Tape, as shown in Figure S14b. It was baked in an oven for one hour at 110 °C and one hour at 160 °C. The resulting surface was not as good as expected. Most of the beams had small circular areas that were not covered with photo resist as shown in Figure S15a. But the layer is good enough to prevent an electrical connection between the cantilever and electrode. The thickness of the insulative layer was measured with a Bruker Dektak Stylus profiler. A scratch was carefully made on the wafer to locally remove the photoresist. A profile measurement was made perpendicular to this line. A typical measurement is shown in Figure S15b. The average thickness over nine points was 1.60 µm (σ = 0.078 µm).

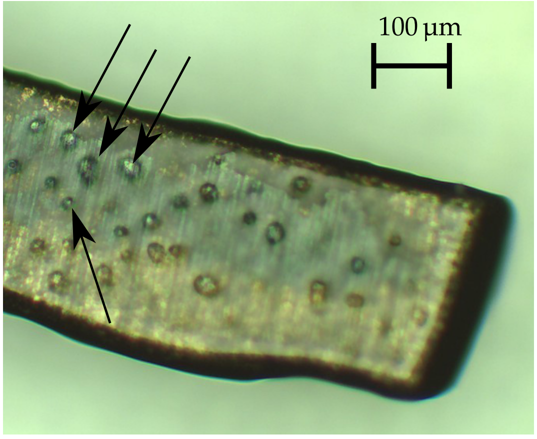

(a) Micrograph of the electrode surface. The layer of photo resist is not homogeneous. Small circular areas appear not to be covered. A few of these areas are indicated with the arrows.

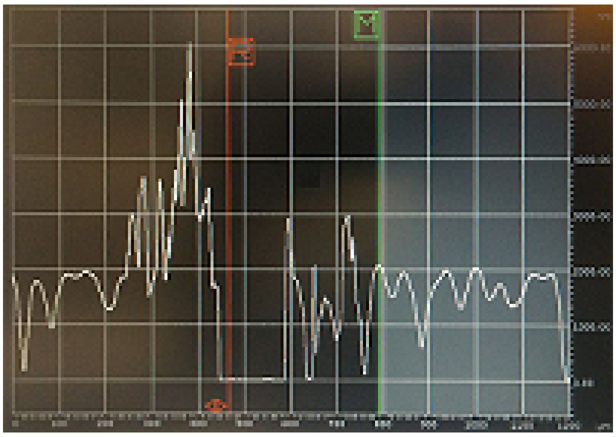

(b) Typical Bruker Dektak profile measurement on the wafer to determine the photoresist layer thickness. A scratch is made in the photo resist and a transverse measurement is done. The red line indicates the location of the scratch, the selected part between 800 and 1200 µm is the where the average value is taken.

Figure S15. Micro graph of electrode surface with photo resist (**a**); Dektak measurement of spray coated photo resist after baking (**b**).

Nanomanipulator

In order to position the electrode relative to the cantilever, a miBot™ robotic nanomanipulator is used. This robot can be positioned in-plane with a minimum resolution of 1.5 nm and a maximum speed of 2.5 mm·s^{-1}. The robot has an arm which can rotate around its actuator. It has a minimum resolution of 0.24 nrad and a maximum rotating speed of 150 mrad·s^{-1} (http://www.imina.ch). The robot is connected to a controller box, which is connected to a computer. An external game controller, that is connected to the computer as well, is used as input device. The electrode is connected to the arm of the robot using double sided non-conductive tape.

Compliant Hinge

The nano-manipulator can vertically position the electrode. The angle of this electrode is uncontrolled though as shown in Figure S16. So in order to be adjust the angle of the cantilever relative to the electrode a compliant hinge was made. The schematic drawing of this compliant hinge is shown in Figure S17. The hinge is made from spring steel and is cut with metal scissors. The holes are punched. The nut is secured with super glue. The metal hinge is placed on a microscope glass with double sided tape. The prototyping PCB is secured with double sided tape as well.

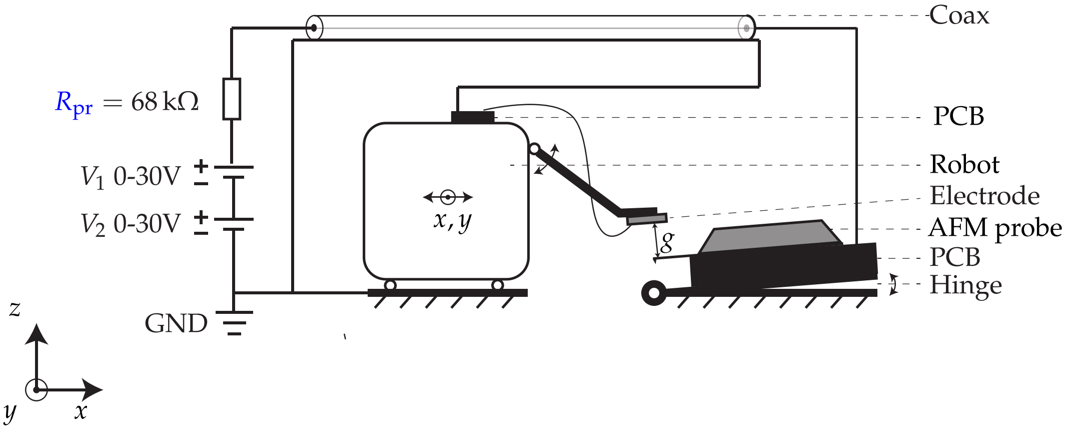

Figure S16. A side view of the experimental setup with the robot, electrode, AFM probe, hinge and electronic components.

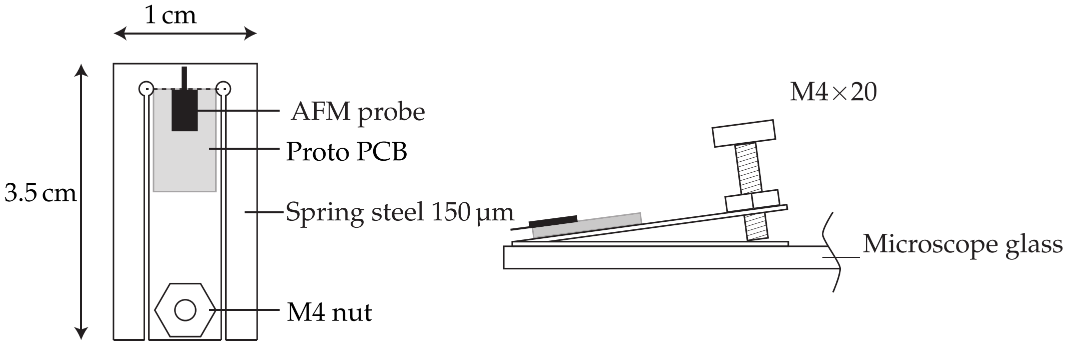

Figure S17. The compliant hinge, used to introduce an extra degree of freedom. The left- and right-side of the hinge is connected to a microscope glass with double sided tape to give it the required stiffness. The PCB is also connected to the hinge with tape. The nut is fixed to the hinge with super glue. By turning the screw, the angle of the cantilever, relative to the electrode, can be adjusted.

Electronics

As a voltage source, two "Delta Elektronika ES 030-5" power supplies are used. They can deliver 30 V each. The voltage sources are connected in series, so a total of 60 V can be obtained. The negative side is connected to the ground, and the positive side to a resistor. This resistor is included for safety measures: If the electrode and cantilever would make electrical contact, the current is limited. It is intended to keep the maximum current though the cantilever below 1 mA, such that the temperature due to Joule heating is limited. This current is common in micro systems, so the cantilever should survive this. The resistance of the circuit itself (R_{circ}) is measured by bringing an electrode without photo resist into contact with the cantilever, and measuring the resistance with a multimeter. This yields 70 Ω. So in order to have a maximum current of 1 mA for the maximum voltage of 60 V:

$$R_{PR} + R_{circ} = \frac{U}{I} \tag{26}$$

$$R_{PR} = \frac{60}{0.001} - 70 = 59\,930\,\Omega \tag{27}$$

The closest available resistance that was a resistor of 68 kΩ.

The electrode is connected to the negative pole of the voltage source and the ground. The electrode is connected to the robot arm with double sided tape. A small piece of prototyping PCB of approximately 5 × 5 mm is connected with double sided tape to the top of the robot. An aluminum wire with a diameter of 30 µm connects the PCB and the electrode. The connection is secured with

conductive silver paint. A wire is soldered to the PCB and connects the voltage source. Because the voltage sources are not directly next to the setup, a coax cable is used to connect the source to the setup. This shields the cables from electric and magnetic fields from outside, and provides a clean and compact connection.

Microscope

A Motic microscope is used to monitor the system and to take images from the deflecting cantilever. The "Motic Image Plus 2.0" software is used to acquire screen shots of the system. The software is calibrated with a calibration slide, to determine the size of the pixels for the different objectives. The objective of the microscope points on top of the cantilever. The cantilever is deflecting perpendicular to the optical axis. In order to be able to observe this, a 45° mirror is used, which is placed close to the cantilever.

Integration of Components

The integration of the components is shown in Figure S18. The nano manipulator is on a metal 'arena', in order for in plane actuator to work at its best. The robot has a flat ribbon connector on the back, that goes to an adapter which is connected to the controller box. The coax connector is secured with tape on the microscope stage, to prevent pulling on the wires during measurement. The cantilever is glued with conductive silver paint to a small plate of prototyping PCB with a conductive top layer. The wire that goes to the voltage source is soldered to the same plate.

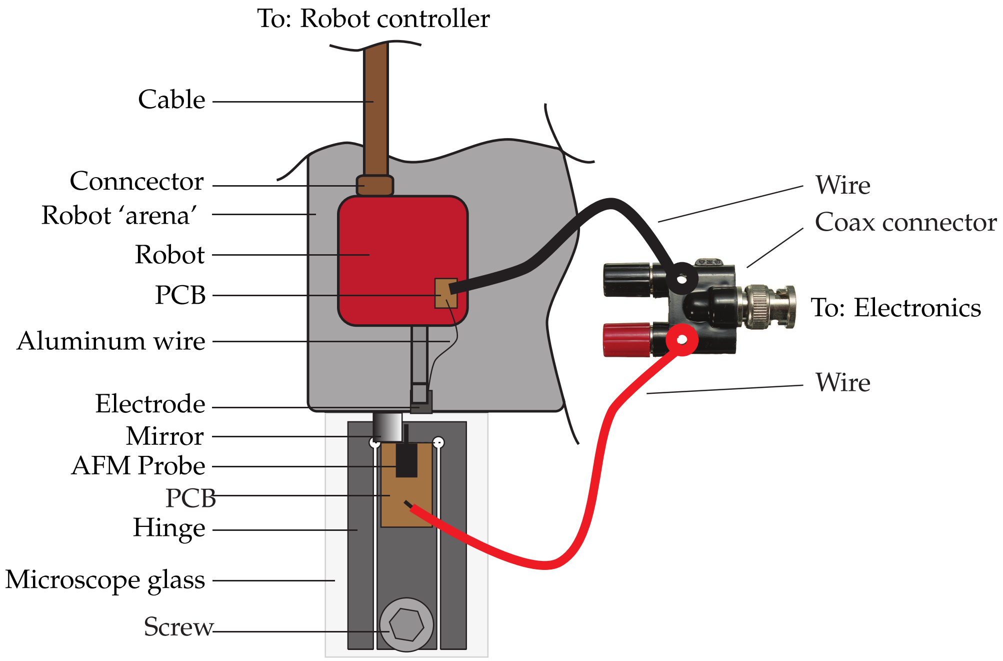

Figure S18. A top view of the experimental setup with the main components. The microscope has the same view on the setup as the view in this figure. The electronics are shown in Figure S16.

Measurement Method and Data Processing

The goal of this experiment is to measure the voltage-deflection behavior of the cantilever to confirm the validity of the models. The measurement data is gathered as follows:

- Make sure that all the components are connected correctly and that the equipment is switched on.
- Manually bring the robot close to the AFM probe.

- Make sure that the electrode is above the cantilever. Use the miBot controller to bring the electrode close to the cantilever. And align the electrode with the cantilever in top-view. Approximate alignment is sufficient for this step.
- Go to side-view of the cantilever by looking through the mirror. Lower the electrode and approach the cantilever to ∼50 µm.
- Focus on the cantilever. Use the in plane motion of the robot to bring the edge of the electrode to the same focal plane.
- Rotate the camera of the microscope, such that the electrode is horizontally aligned on the computer screen. The grid in the microscope software can be used as a reference.
- Raise the arm of the robot such that the electrode is far away from the cantilever.
- Use the compliant hinge to bring the cantilever in a horizontal position relative to the grid, such that it becomes parallel to the electrode.
- Bring the electrode to the required position for the experiment, and verify if the cantilever and electrode are parallel and horizontal.
- Apply the required bias voltage with the voltage sources and make screen shots with the microscope software. Increase the voltage to the next value and repeat until the cantilever is pulled-in.

A number of images is collected, that correspond to a known, varying, bias voltage. A typical measurement is shown in Appendix B. The next step is to post-process these images, to get the voltage-cantilever deformation relation. This was done with Matlab. The basic idea is to compare the images of the cantilever under a bias voltage with the 0 V-image. An optical displacement technique, developed by Kokorian *et al.* [10] was used to achieve sub-pixel resolution displacement measurements.

Optical displacement measurement method The goal of the measurement is to track the measurement point as a function of the bias voltage. This is done by converting the image to gray scale, and looking at the pixel intensity profile along the direction of motion. This is illustrated in Figure S19. This method uses the fact that the shape of this intensity profile remains constant while the cantilever deflects. The shape of the intensity profile is compared for the subsequent images.

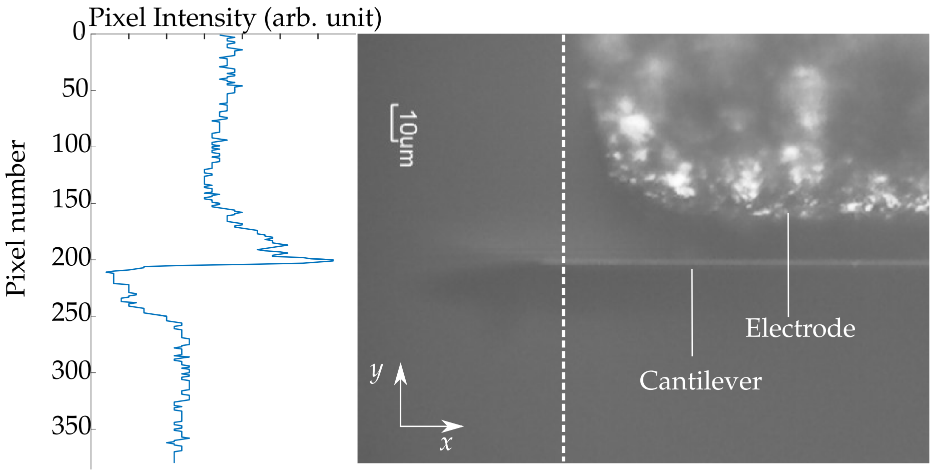

Figure S19. The converted gray scale image with the pixel intensity profile along the dashed line. When the cantilever will deflect, the shape of this peak will remain the same, but the position will change.

First, the intensity profile is averaged for 10 pixels. Then a mathematical function $s(x)$ is generated to describe the peak of the cantilever at 0 V. This is the reference image for the measurement. A spline-based template function is used; this is a numerical function with piecewise polynomial

functions. These functions are connected in the "knots". In between these knots, a cubic line is fitted. The derivatives of two segments in one knot are equal, such that a smooth function is generated. In order to determine the deformation, the intensity profile of the next image is compared with a shifted version of the spline $s'_{x_0} = s(x - x_0)$, where x_0 is chosen such that the spline fits the new intensity profile. In Figure S20 a typical dataset can be found. The original data, and the fitted spline function $s(x)$ are shown. An intensity profile of a deformed cantilever is added to this figure. The shift x_0 is calculated by solving a least square problem:

$$\text{minimize} \sum_{i=1}^{n} \left(s(x_i - x_0) - I_{\text{def}_i}\right)^2 \tag{28}$$

where i is the i-th pixel, x_0 the shift of the spline and I_{def_i} is the i-th pixel of the intensity profile of the deformed cantilever, n is the number of pixels in the intensity profile. The spline shift x_0 can be converted into micrometers using optical calibration of the microscope.

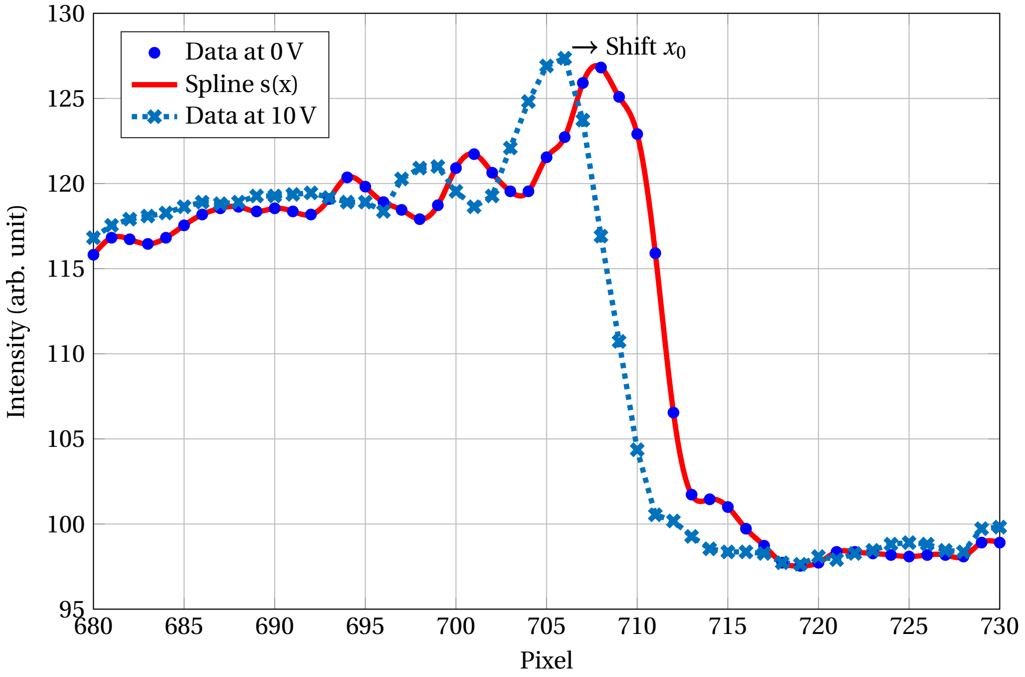

Figure S20. The original intensity profile of the reference image at 0 V, and the fitted spline function $s(x)$ are shown in this figure. The intensity profile data of the deformed cantilever is shown in the same figure. In order to calculate the deformation of the cantilever a least square problem is solved, such that a shifted version of the spline $s'(x_0)$ has the best fit with the new dataset. This x_0 is the amount of pixels that the end of the cantilever has deformed; this value does not need to be an integer, and sub-pixel resolution is achieved.

Measurement error The deflection measurements have a finite accuracy. The error sources are:

- Mechanical error source: The measurement setup is placed on a granite isolation table. This should prevent external vibrations to influence the measurement. It is possible though that mechanical disturbances were transmitted through this isolation table, or that vibrations were induced by the user while performing the measurements. The mechanical loop between the cantilever and electrode is not rigid, which has a negative influence on the measurement. The cantilever and electrode could move relative to each other due to this bad mechanical loop. By measuring the motion of the cantilever, relative to the electrode these error sources are minimized.

- Camera noise: The camera has a limited resolution (3 mega pixels) and is subjected to photon shot noise. This is an error source for the displacement measurement. The pixels of the cantilever and electrode that are used for the measurement are averaged in the horizontal direction, such that optical noise is reduced.
- Optical calibration: In order to convert the displacement of the cantilever in pixels to micro meters, the microscope is optically calibrated with a calibration slide. This calibration is a limit to the resolution of the measurement. The 20× objective that was used for the measurements, was calibrated with a 150 µm circle on the calibration slide. This resulted in 0.3106 $\mu m \cdot px^{-1}$ for the x-direction and 0.3125 $\mu m \cdot px^{-1}$ for the y-direction. The circle is approximately 484 px in diameter and it is estimated that this is at least within ±5 px accuracy. So the confidence interval for the optical calibration is $\frac{5}{484} \cdot 100\% = \pm 1\%$.
- Read-out of voltmeter: A volt meter is used to determine the applied bias voltage. The resolution is limited to two digits up to 20 V and one digit for voltages higher than 20 V.

The largest error source are the optical calibration of the microscope and the resolution of the volt meter. The other error sources will be considered to be negligible compared to these errors. The confidence interval for the bias voltage is ±0.05 V. The confidence interval for the optical calibration is ±1%. The gap between the cantilever and electrode is estimated on pixel-level. It is estimated that this is done within 2 px accuracy, which corresponds with 0.62 µm. The estimate for the gap is used for the models.

2.3.2. Results

The described experiment was performed for different configurations. A total of 16 datasets were collected. Two electrode positions were used. And for each position two initial gaps g_0 were tested (this is an approximate gap, because the exact gap can only be determined in the post-processing step). For each of these configurations four data sets were collected. These measurements are summarized in Table S4, where the pull-in deflection and voltage are given. The voltage-deflection behavior for the datasets are compared with the analytical- and COMSOL-model in Figures S21–S24. Typical micrographs that were collected are shown in Figure S25. A full data set of micrographs is shown in Appendix B.

Table S4. The results of the experiments. The maximum deflection before pull-in d and the corresponding voltage V are given for the measurements (subscript "meas") and model (subscript "mod"). The difference between measurement and model are indicated for the maximum deflection and voltage. This difference is normalized for the value found in the model. and the averages are expressed as a percentage.

L_{eff}	$g_{0\text{-avg}}$	g_0	d_{mod}	d_{meas}	V_{mod}	V_{meas}	Δd	$\frac{\Delta d}{d_{mod}}$	$\left(\frac{100\% \cdot \Delta d}{d_{mod}}\right)_{avg}$	ΔV	$\frac{\Delta V}{V_{mod}}$	$\left(\frac{100\% \cdot \Delta V}{V_{mod}}\right)_{avg}$
50	8.00	8.034	4.31	3.35	30.82	24	0.96	0.22	15%	6.82	0.23	11%
		7.959	4.26	4.04	30.41	33	0.22	0.05		−2.59	−0.07	
		7.650	4.07	3.52	29.00	26	0.55	0.14		3.00	0.11	
		8.350	4.43	3.56	32.80	28	0.87	0.20		4.80	0.16	
	11.29	11.525	6.16	4.81	52.38	46	1.35	0.22	17%	6.38	0.12	9%
		11.213	5.97	5.14	49.33	49	0.83	0.14		0.33	0.02	
		10.900	5.81	4.73	47.40	42	1.08	0.19		5.40	0.13	
		11.525	6.13	5.38	51.29	48	0.75	0.12		3.29	0.08	
75	8.48	8.088	4.74	4.11	35.47	32	0.63	0.13	19%	3.47	0.11	13%
		8.713	5.09	3.95	39.38	35	1.14	0.22		4.38	0.12	
		8.088	4.74	3.69	35.47	31	1.05	0.22		4.47	0.14	
		9.025	5.26	4.34	41.38	36	0.92	0.18		5.38	0.14	
	10.20	10.588	6.17	5.33	51.86	46	0.84	0.14	16%	5.86	0.13	12%
		9.963	5.80	4.61	47.58	43	1.19	0.21		4.58	0.11	
		9.963	5.80	4.95	47.58	43	0.85	0.15		4.58	0.11	
		10.275	5.99	5.18	49.70	45	0.81	0.14		4.70	0.11	

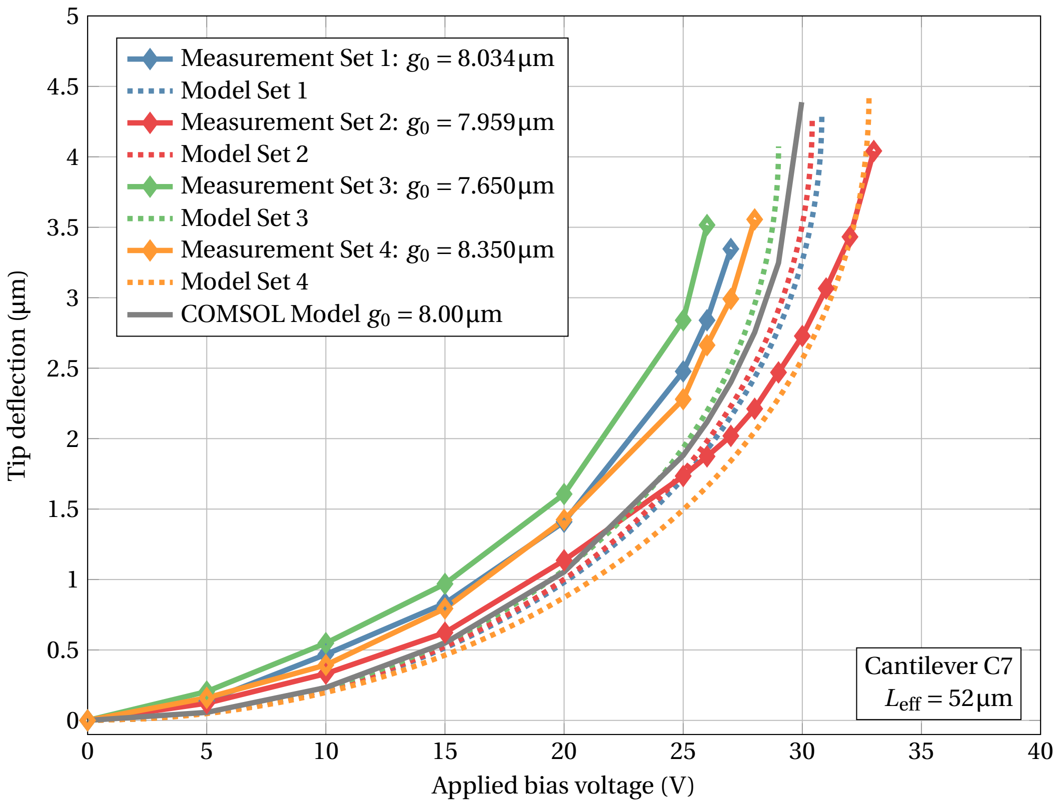

Figure S21. Voltage-deflection behavior of typical measurement.

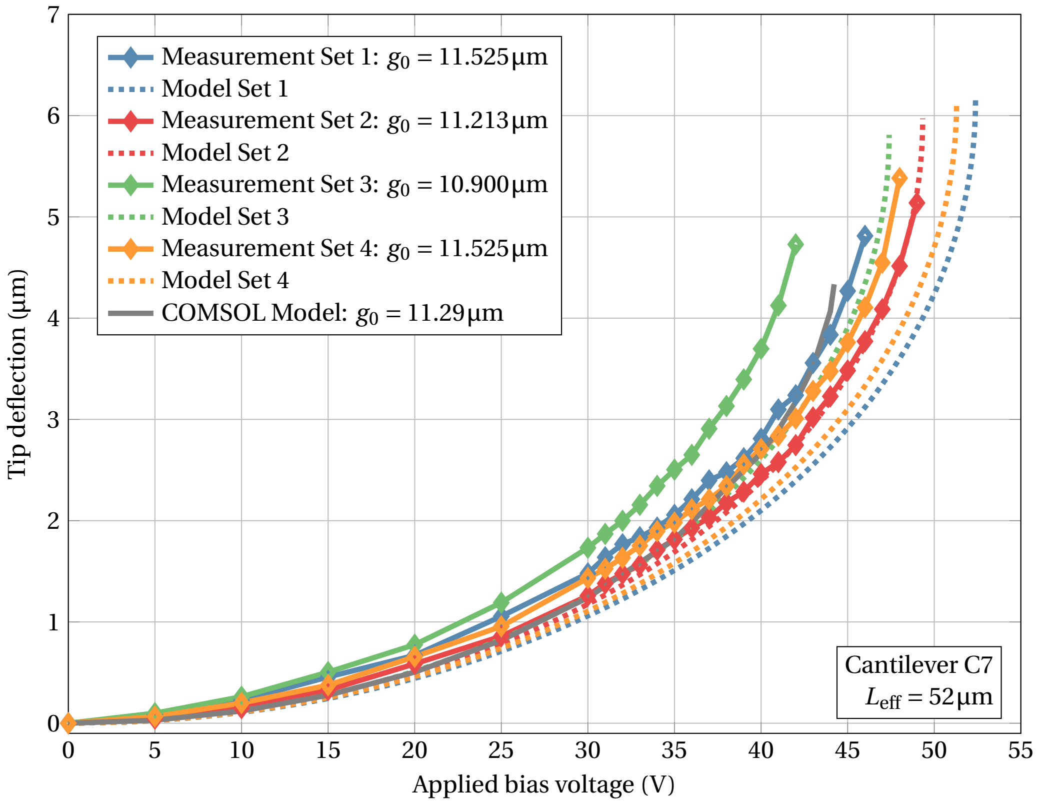

Figure S22. Voltage-deflection behavior of typical measurement.

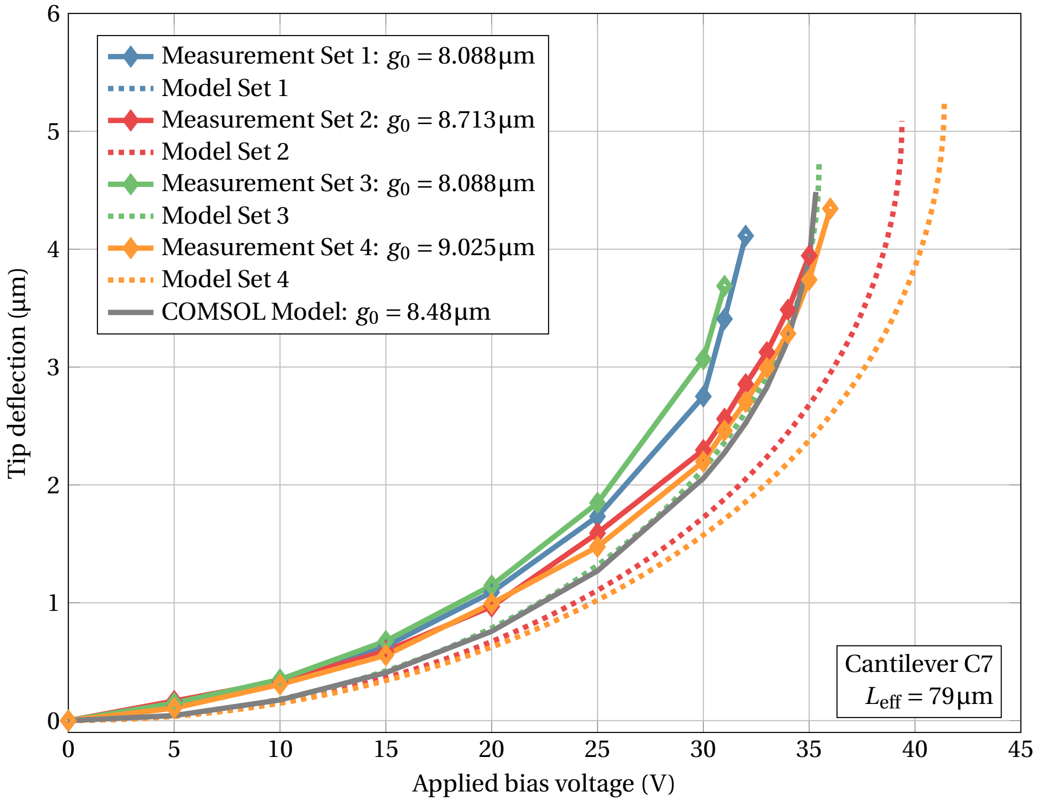

Figure S23. Voltage-deflection behavior of typical measurement.

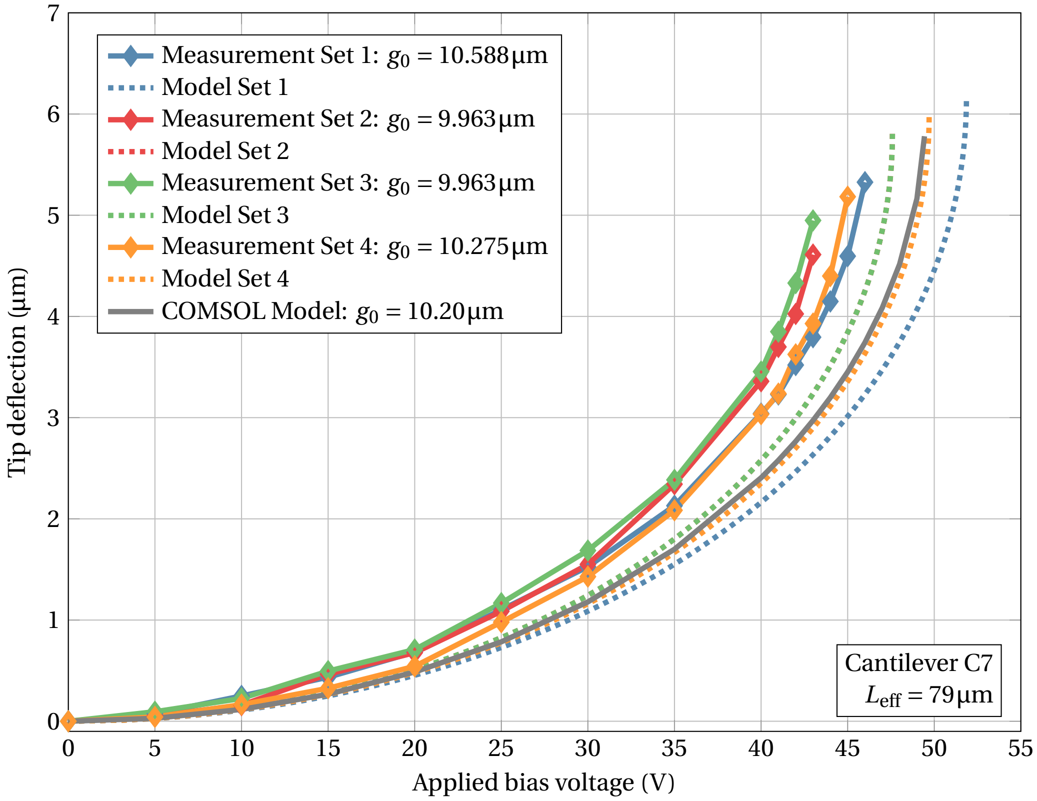

Figure S24. Voltage-deflection behavior of typical measurement.

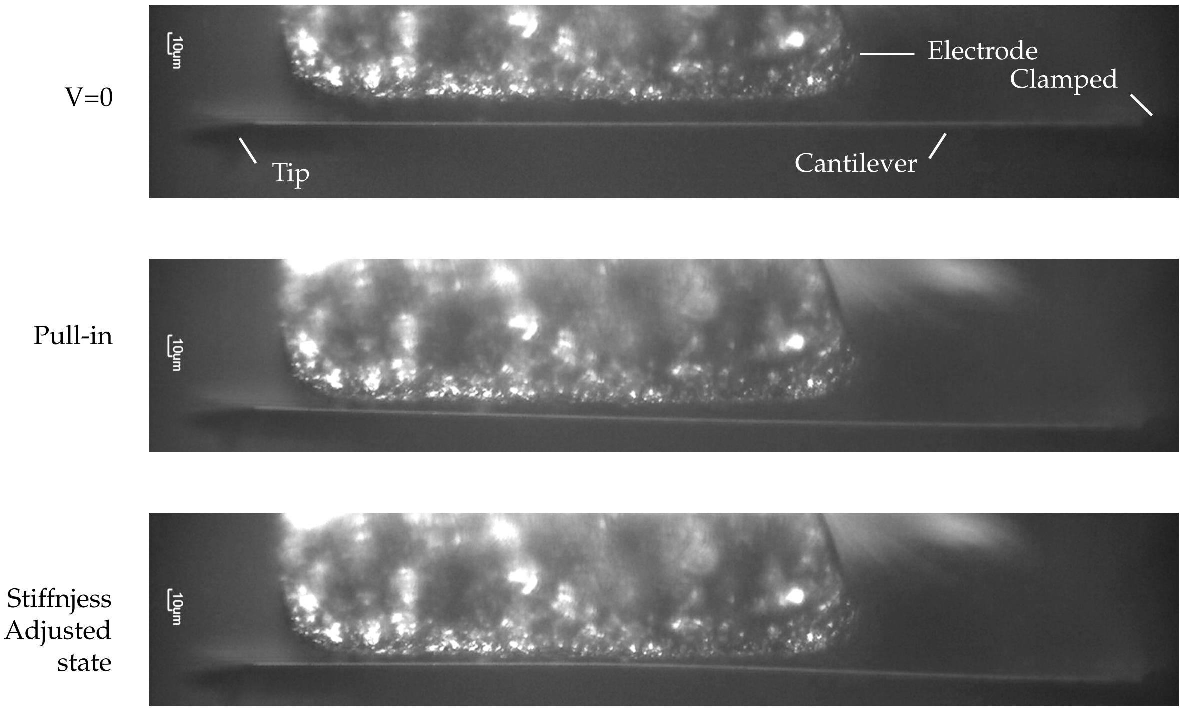

Figure S25. Micro graphs of the cantilever under varying bias voltage. For $V = 0$ the cantilever is in neutral state. When $V = V_{\mathrm{PI}}$ the cantilever is pulled-in to the electrode. When the voltage is increased further, the cantilever will be in adjusted state.

2.3.3. Discussion

In Figures S21–S24 the voltage-deflection behavior of the cantilever under an electrostatic load is compared for the models and measurements. For each data set, the measurement is compared with the analytical model, in the same configuration. For each set of four measurements, the COMSOL model is showing the average configuration. For all of the measurements (except for set 2, Figure S21), the deflection for a given voltage is higher for the measurement compared to the analytical model. So the analytical model underestimates the electrostatic force of the measurement. There are two possible causes for the difference between the analytical model and the measurements. In Section 2.2 it was already concluded that the analytical model underestimates the electrostatic force compared to the COMSOL model, because not all the fringe fields are taken into account. Secondly, the shape of the cantilever is approximated by a constant cross section, as was discussed in the introduction of Section 1. This simplification might lead to an underestimation of the electrostatic force.

There are some other causes that explain the differences between the models and measurements. In the models it is assumed that the electrode is perfectly flat, and that it has sharp edges. In the experimental setup, the edges of the electrode are slightly rounded. It is assumed that those rounded edges do not contribute to the electrostatic force, while they might be of significance.

In Table S4 the pull-in deflection and corresponding voltages are shown. The pull-in deflection of the model is on average 17% higher than the measurement and the pull-in voltage is 11% higher. The measurement is performed in discrete steps of 1 V. So the actual point of pull-in for the measurement is somewhere between the last measured voltage and 1 V higher. The measurement always underestimates the pull-in voltage. When the system is close to pull-in, a small change in bias voltage causes a large change in deflection. So the underestimation of the deflection at pull-in has a significant influence on the measured pull-in point.

It was observed that when the cantilever pulled-in to the electrode, that it did not end up in the expected configuration. There is a intermediate stable state, where the cantilever is touching the electrode, as shown in Figure S25. The voltage needs to be increased beyond the pull-in voltage that

was found with the analytical- and COMSOL model to reach the desired state. In order to investigate this behavior, a time dependent COMSOL model was developed, to model the behavior after pull-in. This is further elaborated in Section 2.4.

2.3.4. Conclusions

The analytical model can predict the voltage-deflection behavior and pull-in voltage. In all measurements there was an average error of 11% in pull-in voltage and 17% in deflection before pull-in. These differences can be explained by the fact that the measurement is performed in steps of 1 V, and will therefor always underestimate these values. Secondly, the analytical model is underestimating the electrostatic force, most likely due to the fact that not all the fringe fields are taken into account and the cantilever geometry is simplified.

The shape of the electrode is not perfectly flat. The edged are not well defined, and the surface quality is not so good. This introduces effects, that are not included in the model. In order to be able to draw definite conclusions about the model, the experiment should be repeated for a more ideal electrode. The electrode should be perfectly straight and have a well defined edge. This could be achieved by micro machining.

The cantilever does not end up in the required state after pull-in. The voltage needs to be increased beyond the pull-in voltage. The models that were developed in this section give insight into the behavior of the system up to the pull-in voltage. Further investigation on post pull-in behavior should be performed. This is done in Section 2.4.

2.4. COMSOL Model: 3D Time Dependent Study

In order to gain more insight into the behavior on how the cantilever pulls-in to the electrode, a time dependent simulation was performed. The goal of the simulation is to see how the cantilever is pulled-in to the electrode. This model is based on 'Pull-in of an RF MEMS Switch' of the COMSOL library.

2.4.1. Modeling

The contact between the cantilever and electrode is modeled as a penalty based force, that rapidly increases when the gap between the electrode and cantilever approaches zero. Only half of the cantilever is modeled, using a symmetry plane in the middle of the with of the cantilever. This reduces the computation time. The model is shown in Figure S26. The mesh which represents the air between the cantilever and electrode is compressed when the cantilever is pull-ed in. This mesh is collapsed into a layer, which has the same thickness as the insulative layer. The insulative layer is not explicitly defined, but is formed by this collapsing mesh. The dielectric constant of this domain is equal to that of air, and rapidly approaches the value of the insulative layer, when the mesh is compressed. The contact between cantilever and electrode is modeled with a penalty based method [11]. The force F_c increases rapidly, when the gap g between cantilever and insulative layer approaches zero. The sign of the force changes, when the gap is smaller than, or equal to zero:

$$F_c = t_n - e_n \cdot g \qquad g \leq 0 \tag{29}$$

$$F_c = t_n + \exp\left(-\frac{e_n}{t_n} \cdot g\right) \qquad g > 0 \tag{30}$$

With e_n the penalty stiffness and t_n an input estimate of contact force. The voltage is applied in a smooth step, going from zero to the set voltage in 1×10^{-5} s. The simulation is performed with a time dependent study, ranging from $0 - 15 \times 10^{-5}$ s in steps of 2.5×10^{-7} s. For this configuration, the pull-in voltage is 20.5 V, according to the analytical model. Up to the pull-in voltage the system is studied with a stationary study. Beyond pull-in a time dependent study is performed. The stationary

study is performed from zero to pull-in voltage in steps of 1 V. The time dependent study is done in steps of 2 V between the pull-in voltage and 65 V and steps of 5 V up to 85 V.

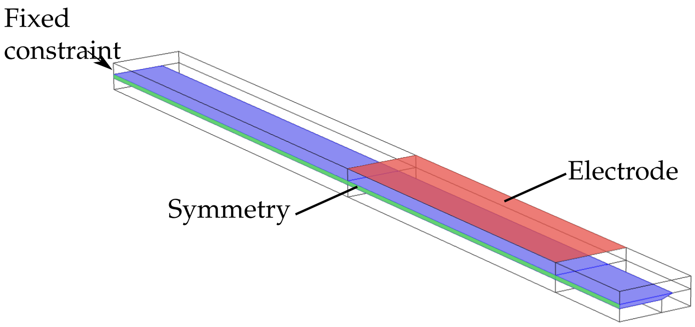

Figure S26. Geometry of the COMSOL model for time dependent simulation.

Mesh

The mesh is based on the original model. A quad mesh is applied to the cantilever, and the air at the bottom side as shown in Figure S27a. This mesh is swept trough the geometry as shown in Figure S27b. The top surface of the cantilever is swept to the top boundary of the geometry as shown in Figure S27c.

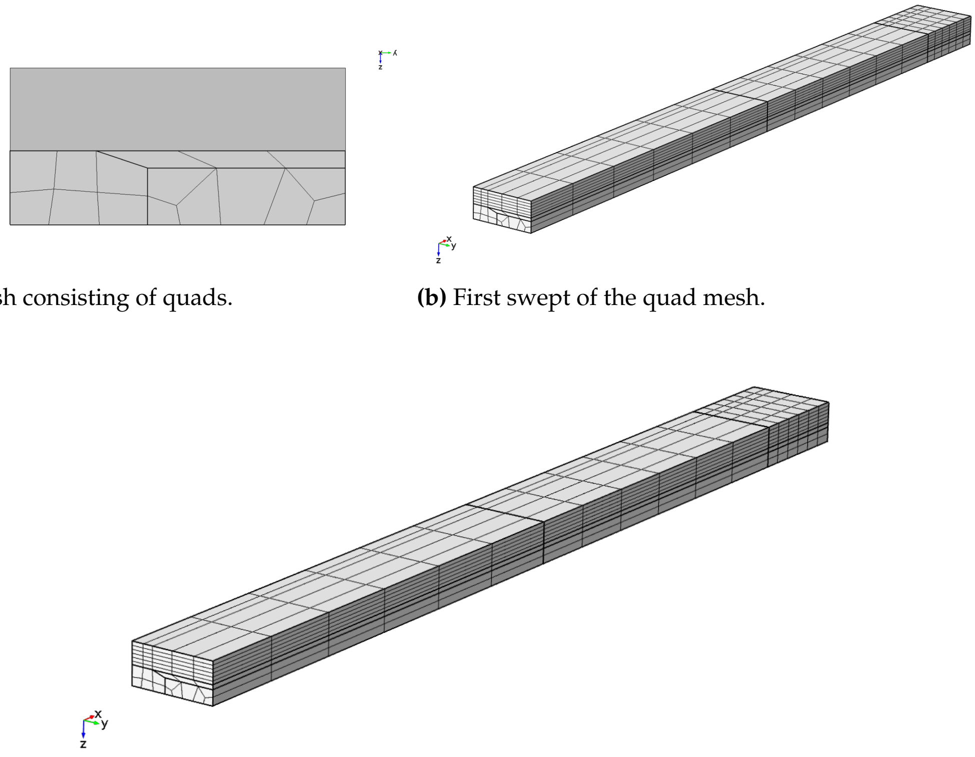

(a) Mesh consisting of quads. **(b)** First swept of the quad mesh.

(c) Swept of the air gap between cantilever and electrode.

Figure S27. Mesh of the time dependent COMSOL model.

2.4.2. Results

In the article the main results of the model are shown. The shape of the cantilever and tip displacement were shown as a function of applied bias voltage. Additional results are shown in this section. In Figure S28, the tip deflection is shown as a function of time. This is compared for a voltage before pull-in, right after pull-in and beyond pull-in. For voltages higher than pull-in, the damping is higher, due to the contact with the electrode. But for voltages smaller than pull-in, the cantilever keeps oscillating and is only lightly damped. Therefor a static analysis is performed up to pull-in voltage (0–21 V). In Figure S29 the final, stable states for 35 and 80 V are shown, which are before, right after and way beyond the pull-in voltage of 20.5 V.

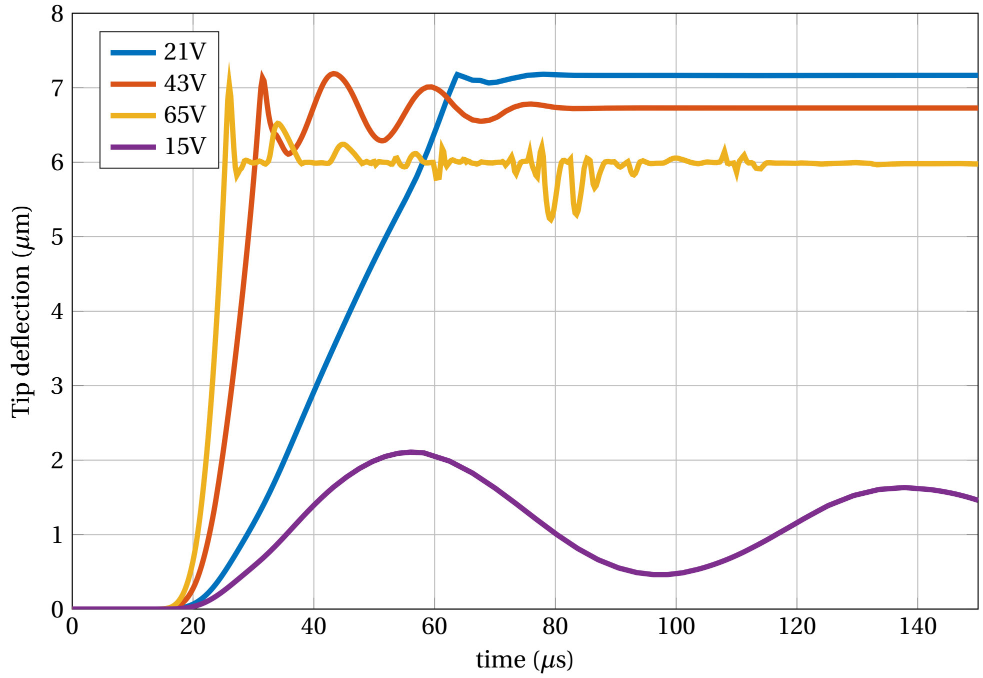

Figure S28. Tip deflection for a varying voltage as a function of time.

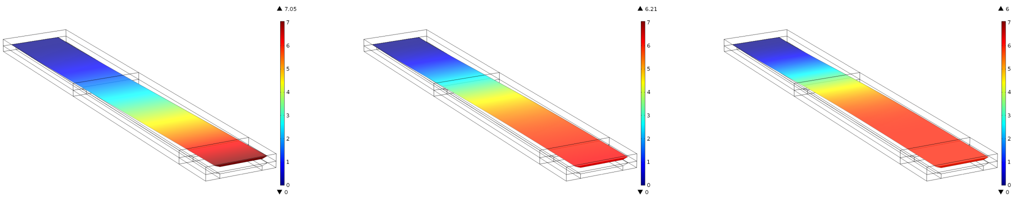

(a) Deflected 3D COMSOL model in steady state for 30 V **(b)** Deflected 3D COMSOL model in steady state for 55 V **(c)** Deflected 3D COMSOL model in steady state for 80 V

Figure S29. The results of the deflection in the 3D format is shown for (**a**) 30V (**b**) 55V and (**c**) 80V.

2.4.3. Discussion

The pull-in voltage that was found for the time-dependent study was smaller than the one found for the stationary study. In the stationary study, the system finds the maximum deflection where it is still possible to find equilibrium. For any given voltage below pull-in in the time dependent study, the cantilever overshoots the final position that it would have in the stationary study. This is due to inertia. For the stationary study pull-in is at approximately 22 V, for the time dependent at approximately 21 V.

3. Supplementary Material: Dynamic Mode Characterization of Stiffness Change

In this chapter the details of this experiment are presented. In Section 3.1 the experimental setup is discussed. This is followed by obtained results in Section 3.2, which are additional to the ones presented in the main article.

3.1. Experimental Setup

The experimental setup was presented in the main article. In this section more details about the setup are shown, as well as on how the measurements were conducted.

3.1.1. Piezo Actuation of Cantilever

The cantilever was placed on a stack of a piece of prototype PCB, the piezoelectric actuator and a sheet of 125 µm PDMS from Shielding Solutions. The piezoelectric actuator is manufactured by Piezo Systems Inc. (Massachussets, USA) and is cut from a large sheet of 0.005 in thick. It was clamped by a small mechanical spring. This spring is secured to the rest of the stack with tape. The stack is placed on the compliant hinge, that was shown in detail in Section 2.3.1 This stack is shown in more detail in Figure S30.

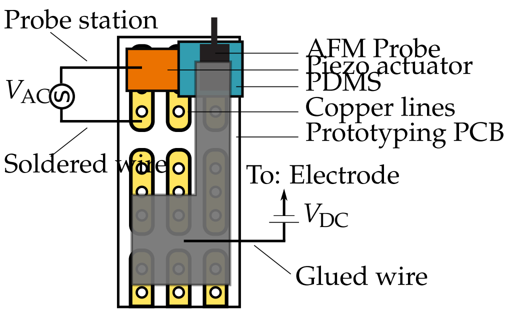

(a) Schematic of stack.

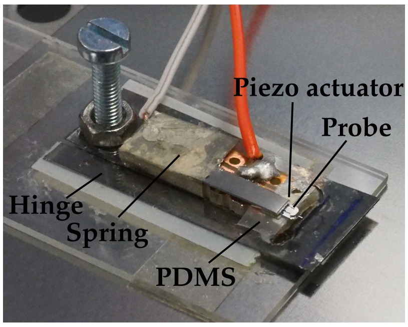

(b) Photo of stack.

Figure S30. Stack of a piece of prototyping PCB, with the piezoelectric actuator, PDMS, cantilever and spring. The electrical connections are indicated.

3.1.2. Laser Doppler Vibrometer

The experimental setup is shown in Figure S31. The system was placed in a Polytec-MSA-400 laser Doppler vibrometer. The system uses the Doppler effect to detect vibrations. The system sends a laser beam through a microscope objective. The beam can be deflected by the scan head, such that multiple measurement points can be taken automatically. The reflection of the laser used to measure the Doppler shift, which is a measure for the velocity of the vibrating sample. The internal signal generator of the system was used to drive the piezoelectric actuator. This signal was also used as a trigger for the system, by connecting the "sync" port to the "trigger in" port. This makes sure that a new measurement starts synchronous with a new input signal. A "burst chirp" signal was used with frequencies from 1 kHz to 500 kHz and a voltage of 10 V. The measurement points were averaged 10 times (Complex averaging), the bandwidth was set from 0 kHz to 500 kHz. The velocity of the vibrometer was set to 25 mm·s^{-1}·V^{-1}, such that it can handle the large bandwidth.

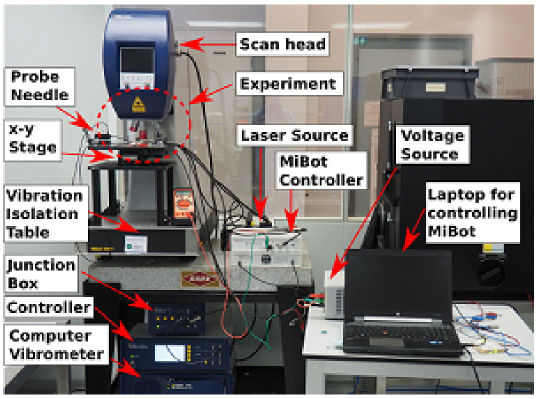

(a) Photo of measurement setup.

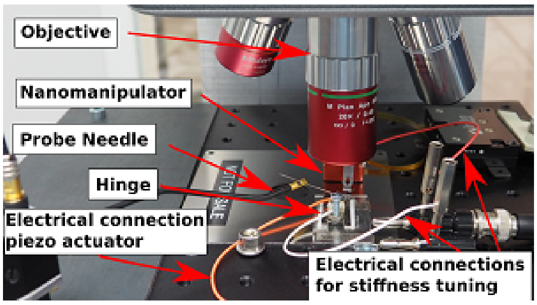

(b) Close up of measurement setup.

Figure S31. Experimental setup for vibration measurements.

3.1.3. Measurement Procedure

The measurement procedure is similar to the one used for the voltage-deflection experiment of Section 2.3.1. The steps of a measurement:

- Manually position the nanomanipulator close to the probe.
- Make sure that the electrode is higher than the probe
- Use the controller of the nanomanipulator to bring the electrode close to the probe. The electrode should be aligned from the top view
- Go to the side view, by using the x-y-stage and focus on the cantilever.
- Bring the electrode close to the cantilever and bring it into focus by moving the nanomanipulator in plane.
- Check if the cantilever is perfectly parallel by comparing the cantilever before and after pull-in. The software tool "Meazure" was used to mark the position of the cantilever before and after pull-in. (see Figure S32)
- If needed, use the hinge to adjust the angle of the cantilever relative to the electrode.
- Test the pull-in behavior and estimate the last point of contact between electrode and cantilever. By varying the applied voltage, a rotation point can be observed, which is the last point of contact. The effective length is determined according to this point. This point is assumed to be found within ± 10 px.
- Bring the electrode close to the cantilever (~ 5 µm). Choose the right position along the length of the cantilever and measure the effective length. Apply a voltage for pull-in.
- Return to top view by using the stage, focus and choose the measurement points. (see Figure S33). Run the measurement.
- Go back to side view, and repeat the final three steps until all measurement points are completed.

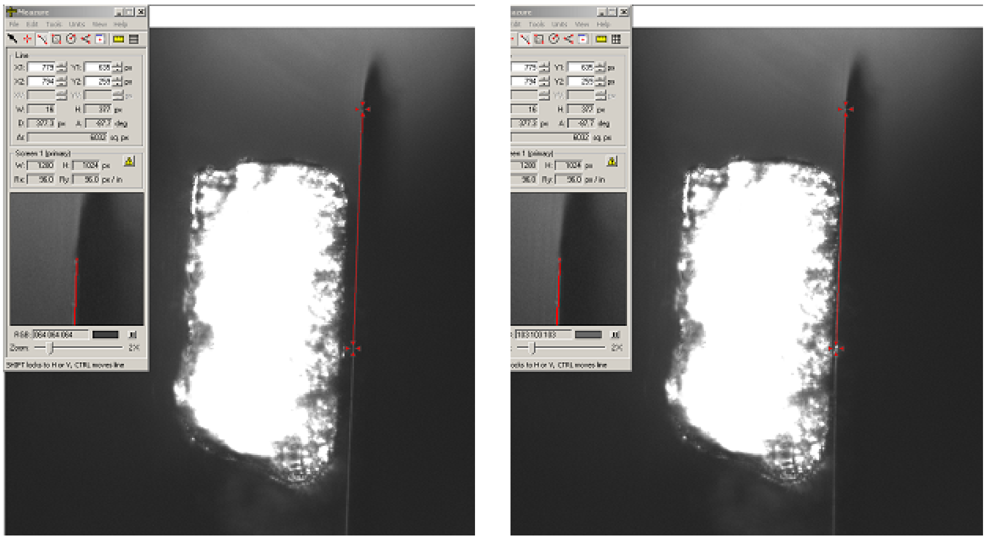

(a) Cantilever in untuned state. The tool "Meazure" is used to indicate the position of the cantilever.

(b) The cantilever is in tuned state. The stage of the vibrometer is used to bring the cantilever to the red line that was drawn with the tool "Meazure", and it is verified that the cantilever and electrode are parallel.

Figure S32. Verification that electrode and cantilever are parallel.

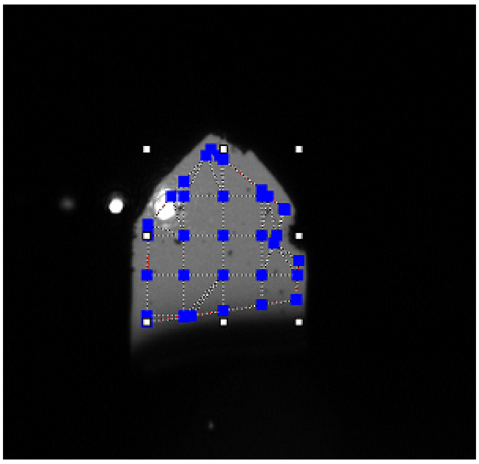

Figure S33. Measurement point on cantilever in tuned state. Only a part of the effective length is visible, due to the shape of the electrode and the shade on the cantilever.

3.2. Results

In this section, additional results are presented. First, the higher order resonance frequencies and modes of a cantilever without adjustments are shown in Section 3.2.1. In the main article, the results of a single measurement were presented. A second data set with similar results is shown in Section 3.2.2.

3.2.1. Higher Order Modes

A measurement was performed to find the higher order modes of the cantilever (Figures S34 and S35). This was done without the electrode in close proximity. The bandwidth of the measurement is 500 kHz.

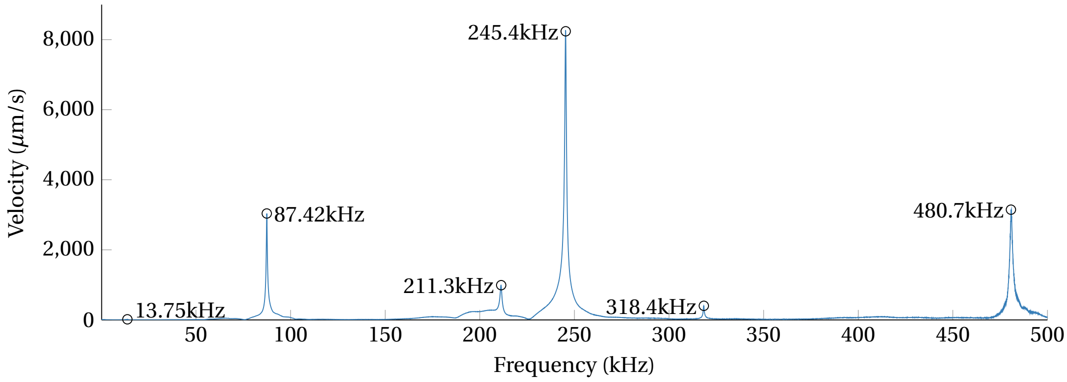

Figure S34. Resonance frequencies of the cantilever for a bandwidth of 500 kHz.

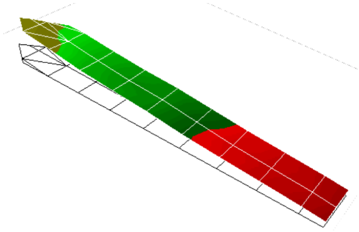

(a) First bending mode 13.75 kHz

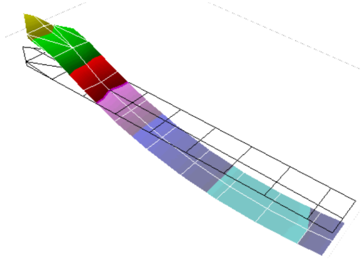

(b) Second bending mode 87.42 kHz

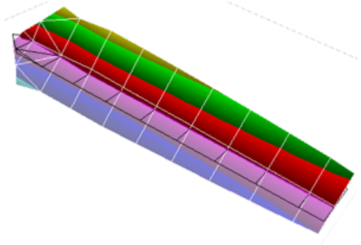

(c) First torsional mode 211.3 kHz

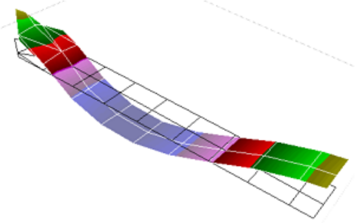

(d) Third bending mode 245.4 kHz

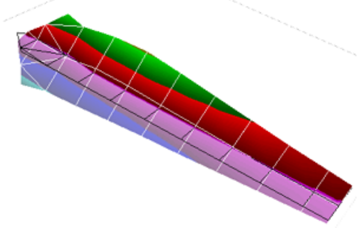

(e) Second torsional mode 318.4 kHz

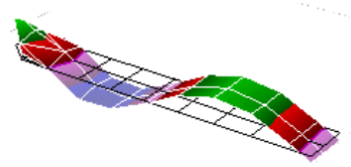

(f) Fourth bending mode 480.7 kHz

Figure S35. Mode shapes of the cantilever, that corresponds with the modes from Figure S34.

3.2.2. Additional Data Set

Inthe main article the results of the measurements were presented. The measurement was repeated, which is shown in Figures S36 and S37.

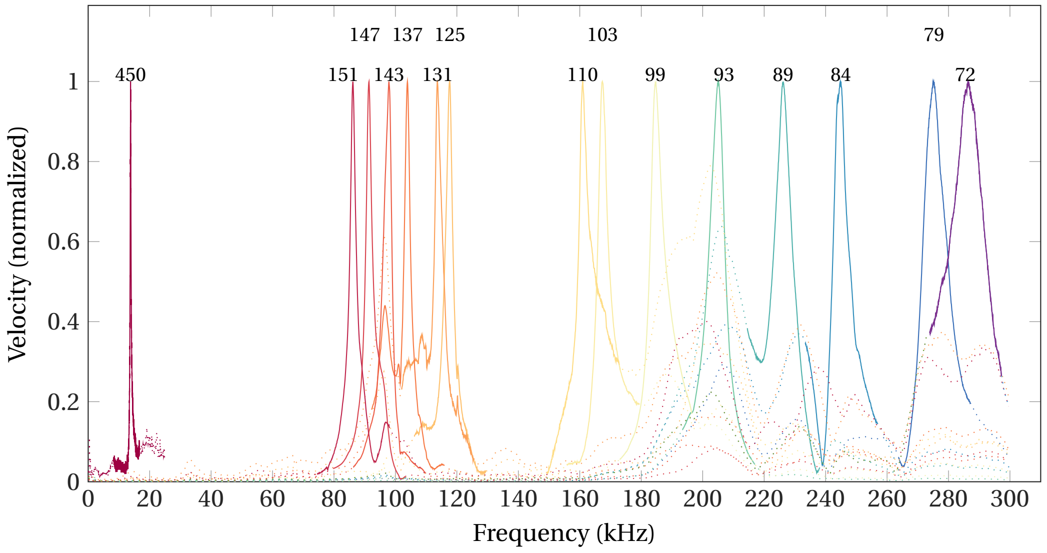

Figure S36. The fundamental resonance frequency obtained for different adjusted lengths of the cantilever. The electrode position is varied and the cantilever is pulled-in for these positions, reducing its effective length. The cantilever is actuated with a frequency sweep and the response is shown in this figure. The numbers above the peaks indicate the effective length of the cantilever (in µm). Only the resonance peaks of the cantilever are shown in a solid line, while the rest of the bandwidth is plotted as a dotted line for clarity. All the measurements were made at an applied voltage of 60 V. This data set is additional to the one shown in the main article.

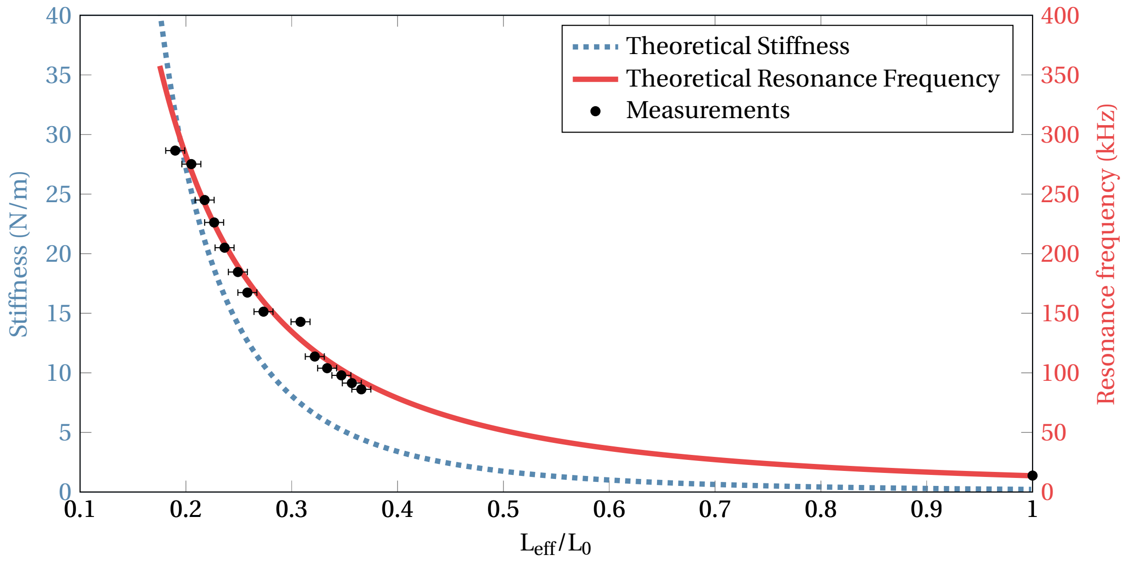

Figure S37. Theoretical stiffness and resonance frequency compared with the measurements as a function of the normalized effective length. The measurement points correspond with the peaks found in Figure S36. The error bars correspond to the measurement uncertainty on the effective length.

3.3. Discussion and Conclusions

The results are very similar to ones shown in the main article. There is one data point in Figure S37 at $L_{\text{eff}}/L_0 \sim 0.3$, which deviates from the expected trend. It is uncertain what has caused this. The behavior of the resonating cantilever at other frequencies than the resonance frequency was not discussed in the main article. Between 80–120 kHz and 180,300 kHz the spectrum is not flat outside of the resonance peaks. It was assumed that this is caused by vibrations in the rest of the system. This was confirmed by performing a frequency response measurement on top of the electrode, which is shown in Figure S38. The non-normalized frequency responses are shown for both the cantilever with varying effective length, and the top of the electrode. There are two regions where the frequency response of the electrode is significant (around 100 kHz and 200 kHz). There are two observations: The resonance peaks of the cantilever become higher when they are closer to these regions. And when there is no resonance peak of the cantilever near these spots of the electrode, the cantilever gives a response. For the region around 100 kHz it is a very small effect, but around 200 kHz it is significant. Considered that these spots are all at the same frequency, and that these correspond with the frequency response of the electrode it can be concluded that this effect is caused by vibrations of the electrode.

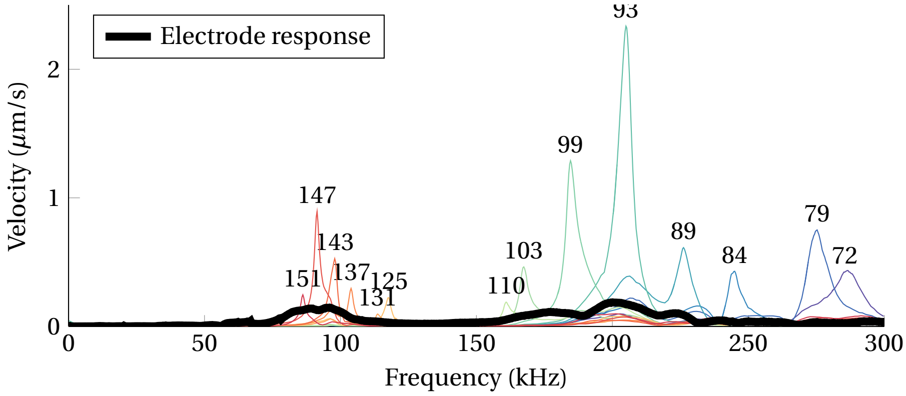

(a) Frequency response of the cantilever for a varying effective length (the numbers near the peak indicate the effective length in µm).

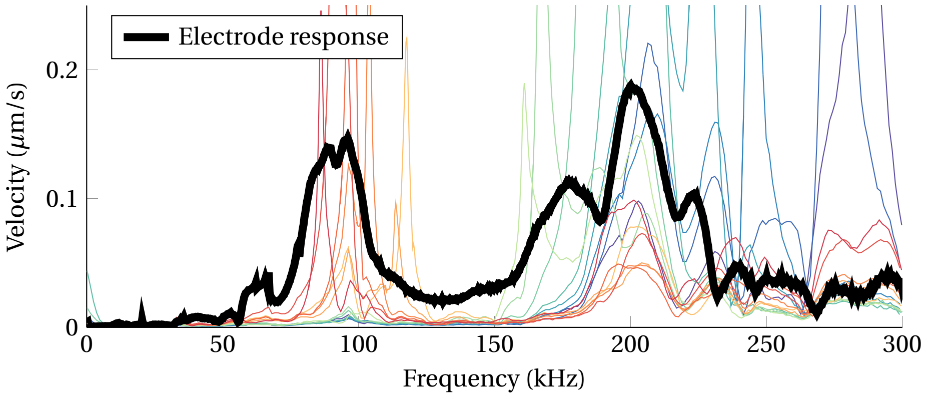

(b) Frequency response, with zoomed in y-axis.

Figure S38. Frequency response of the cantilever and electrode.

3.3.1. Squeeze Film Damping

During the measurement it was observed that when the the cantilever was in unadjusted state, but the electrode was close to the cantilever (∼5–10 µm), the first resonance mode was heavily damped,

while the second mode was apparently unaffected. Squeeze film damping could be the cause of the observed effect. In order to determine if this could be the case, a brief analysis has been performed.

Squeeze film damping is a phenomenon which is often observed in MEMS. When two structures are moving very close to each other, the air film in between is resulting in a lateral flow as shown in Figure S39. For slow speeds, the air can escape between the plates, but the viscous forces of the air give a damping effect. For high speeds, the air cannot escape from the gap. The air is compressed and acts like a spring. This results in a stiffening effect of the system.

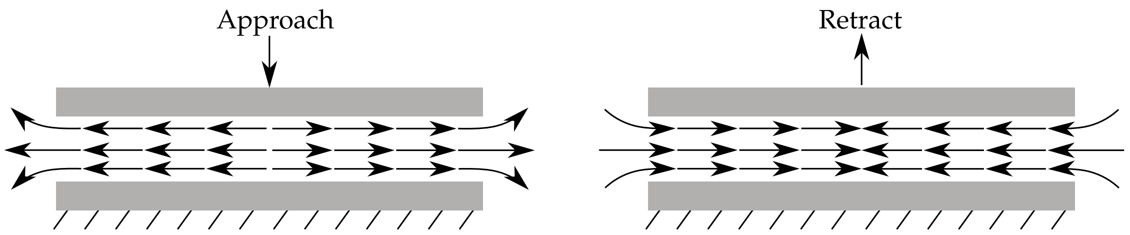

(a) Top plate is approaching. The air is squeezed out. **(b)** Top plate is retracting. The air is sucked in.

Figure S39. Schematic of squeeze film effect.

Pandey *et al.* [12] presented an analytical model that describes the influence of both the resonance frequency and the mode shape of an oscillating micro cantilever on the squeeze film effect. This model was compared with finite element results and experiments. The model predicts the damping ratio ξ within 10% compared to measurements (a low damping ratio results in a high qualtiy factor Q). It was concluded that the damping ratio decreases for an increasing frequency, and that also the mode shape has a significant influence on this damping ratio. In a comparable configuration ($L \times w \times t = 350 \times 22 \times 4\,\mu\text{m}$ and $g_0 = 1.4\,\mu\text{m}$) the damping ratio for the second and third out of plane mode were reduced by 84% and 94% respectively. The model assumes that inertia effects of the squeeze film may be neglected, which is valid when the Reynolds number is smaller than unity. To see if this assumption is also valid for the present system the Reynolds number Re is calculated as:

$$Re = \frac{\rho_a {g_0}^2 \omega}{\mu_{\text{eff}}} \tag{31}$$

where ρ_a the density of air ($1.2\,\text{kg}\cdot\text{m}^{-3}$), ω the angular resonance frequency and the effective dynamic viscosity μ_{eff}, which is a function of the dynamic viscosity μ and the Knudsen number $Kn = \lambda / g_0$ ($\lambda = 65\,\text{nm}$ the mean free path of air):

$$\mu_{\text{eff}} = \frac{\mu}{1 + 9.683 Kn^{1.159}} \qquad 0.01 < Kn < 0.1 \tag{32}$$

The maximum Reynols number is 0.1, for $g_0 = 10\,\mu\text{m}$ and resonance frequency of the second mode $f = 87\,\text{kHz}$. It is concluded that the model which was developed by Pandey *et al.* is valid for the present work, because the assumptions are still valid. So similar results may be expected. The fact that the first order mode is not visible when the electrode is close to the cantilever, while the second order mode seems unaffected can be explained by the squeeze film effect. A more detailed study should be performed before definite conclusions can be drawn.

Appendix A. SEM Micro Graphs Cantilever

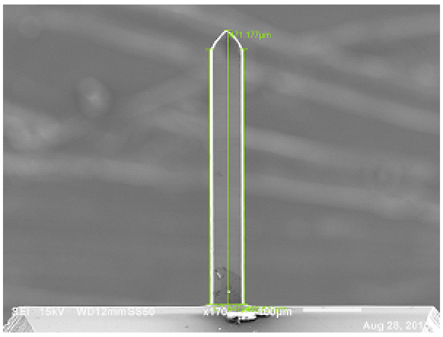

(a) Top of the cantilever to determine the lengths.

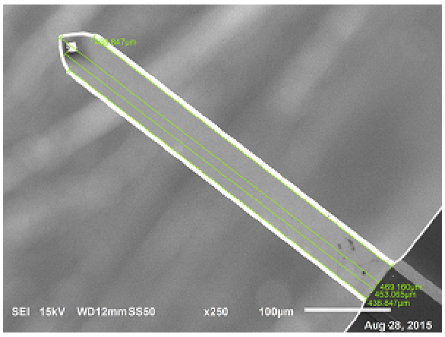

(b) Bottom of the cantilever to determine the lengths.

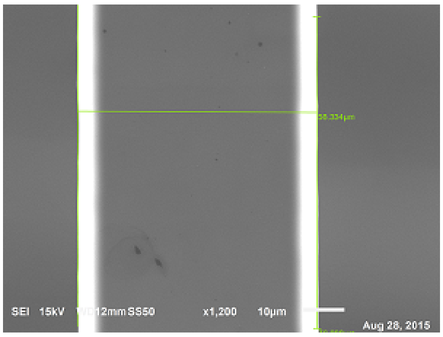

(c) Top of the cantilever to determine the width.

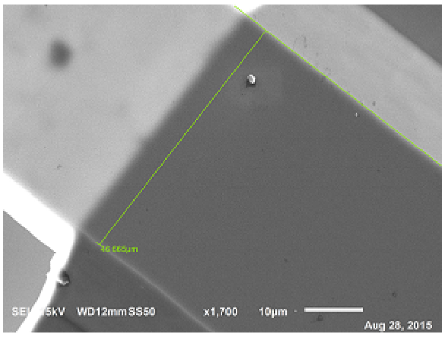

(d) Bottom of the cantilever to determine the width.

Figure A1. SEM images taken from cantilever C1 to determine the dimensions.

Appendix B. Typical Dataset Voltage-Displacement Measurement

The following micrographs are the result of a voltage-deflection measurement, as described in Section 2.3. This dataset is the same as Figure S21. In all the following figures, the fixed side of the cantilever is on the right side of the image.

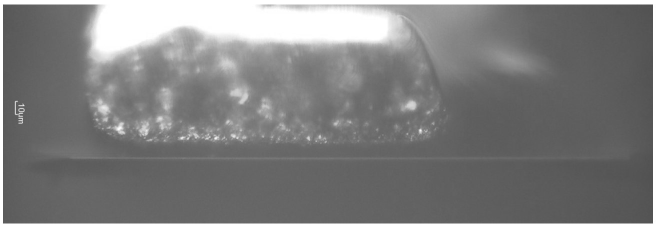

(a) $V_{DC} = 0$

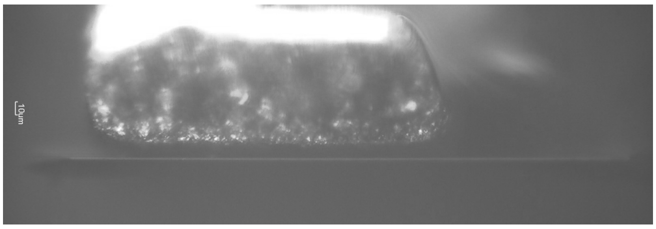

(b) $V_{DC} = 5$

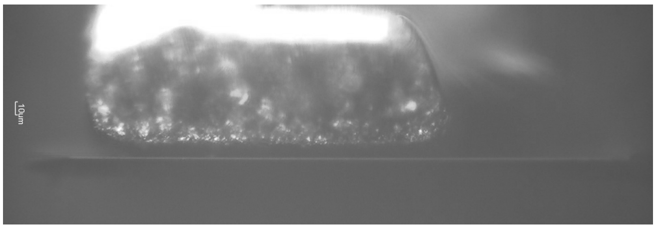

(c) $V_{DC} = 10$

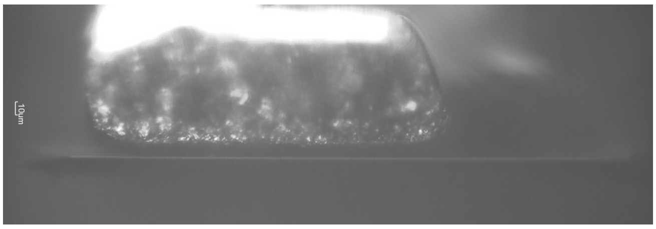

(d) $V_{DC} = 15$

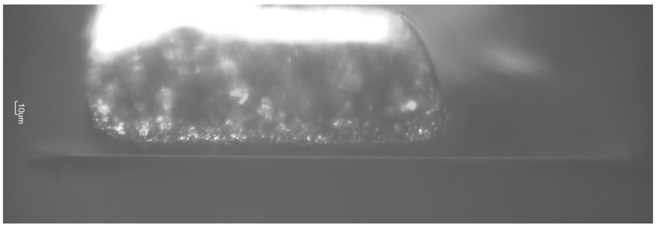

(e) $V_{DC} = 20$

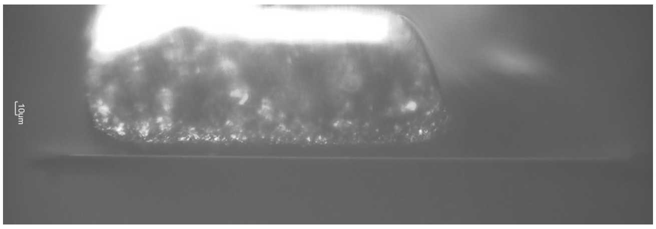

(f) $V_{DC} = 25$

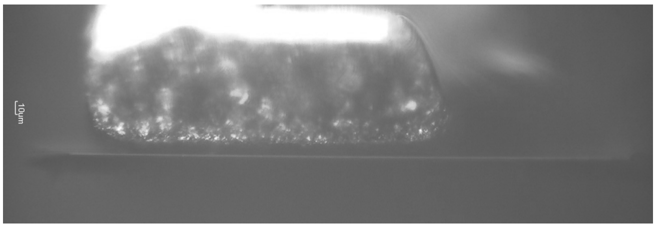

(g) $V_{DC} = 26$

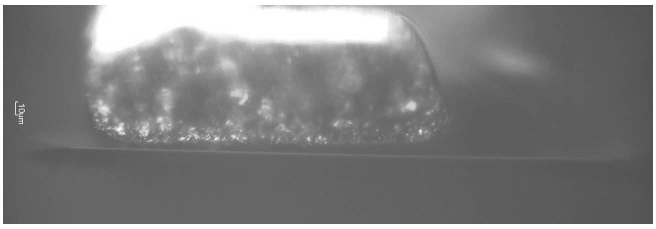

(h) Pull-in: $V_{DC} = 27$

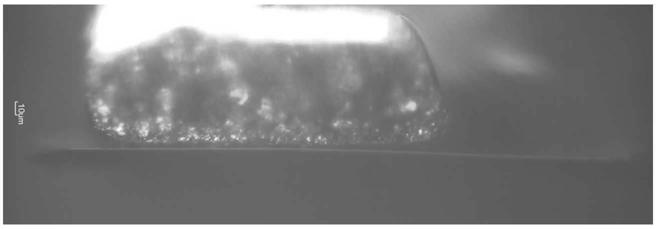

(i) Stiffness adjusted state $V_{DC} \sim 50$

Appendix B.1. COMSOL Model

A 3D COMSOL model has been developed, such that the voltage-deflection behavior can be modeled for the design and the pull-in voltage can be calculated. A 2D model provides insufficient details compared to a 3D model, because the fringe fields and asymmetric electrode as shown in Figure B1 cannot be taken into account in such a model. In this section the model is presented. The details of this model are found in Section 2.

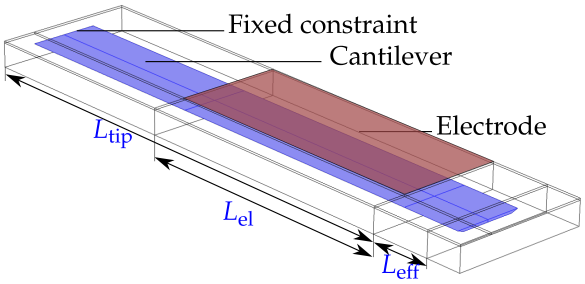

Figure B1. 3D COMSOL model with the cantilever and electrodes indicated.

Appendix B.1.1. Modeling

The geometry of the cantilever is modeled according to measurements that are defined in Section 1.1. The 3D model is shown in Figure B1. The electromechanics physics are used to model the system; the base of the cantilever is defined as a fixed constrained as shown in Figure B1, the electrodes are defined as the electrical ground and the voltage is applied to the entire surface of the cantilever.

In order to determine the pull-in voltage of the system, an inverse problem is solved. Instead of applying a certain voltage and determining the deflection, a deflection Z_{set} is applied to the cantilever (at the tip position), and the required bias voltage for equilibrium is calculated. This method is based on "Pull-in Voltage for a Biased Resonator" from the COMSOL library (www.comsol.com). When the pull-in voltage is found from this study, a voltage-deflection study is performed up to this point. A range of voltages is applied, and the resulting deflection is simulated.

Appendix B.1.2. Results

The result of the simulation is a voltage-set point relation as shown in Figure B2a. The pull-in voltage is at the maximum of this plot; up to this point the system is stable, but after this maximum the system is unstable. Now that the pull-in voltage is known, the voltage-deflection relation is simulated in a separate study, up to the calculated pull-in voltage as shown in Figure B2b. A 3D representation of the cantilever with maximum deflection before pull-in is shown in Figure B3. A 2D plot of the potential in the system is shown for two cross sections in Figure B4.

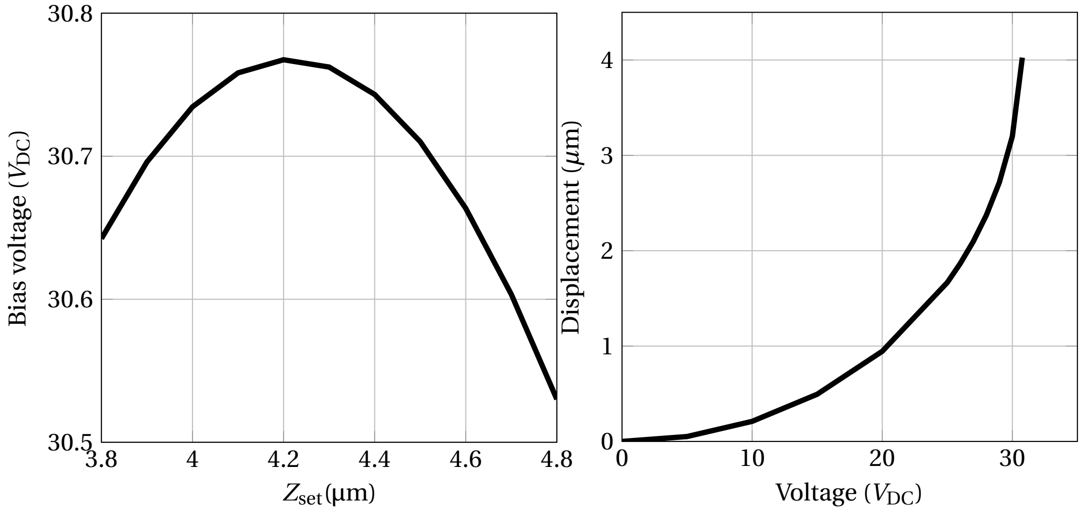

(a) The required bias voltage for equilibrium as a function of the deflection of the cantilever.

(b) Deflection of the cantilever as a function of the voltage.

Figure B2. Results of the COMSOL simulation.

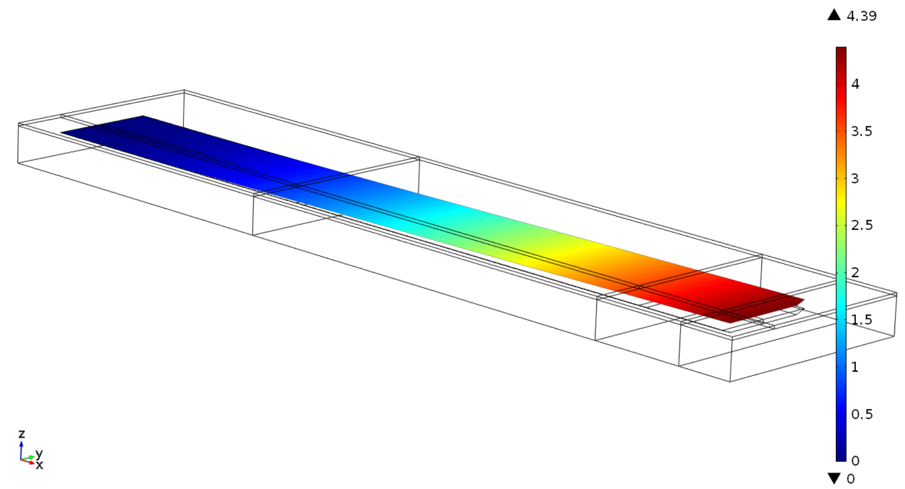

Figure B3. 3D representation of cantilever with maximum displacement before pull-in. Deflection is in µm.

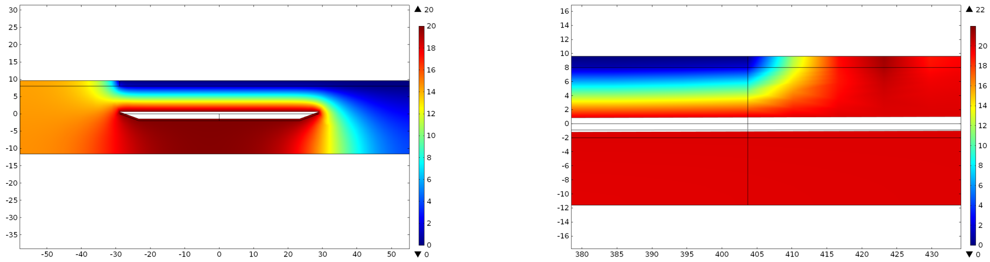

(a) *ZY* plane cut. Along the width of the cantilever. **(b)** *XZ* plane cut. Along the length of the cantilever. Zoomed in near the edge of the cantilever.

Figure B4. Plane cuts from COMSOL results at 20 V.

References

1. Do, C.; Lishchynska, M.; Delaney, K.; Hill, M. Generalized closed-form models for pull-in analysis of micro cantilever beams subjected to partial electrostatic load. *Sens. Actuators A Phys.* **2012**, *185*, 109–116.
2. Hopcroft, M.A.; Nix, W.D.; Kenny, T.W. What is the Young's Modulus of Silicon? *J. Microelectromech. Syst.* **2010**, *19*, 229–238.
3. Bochobza-Degani, O.; Nemirovsky, Y. Modeling the pull-in parameters of electrostatic actuators with a novel lumped two degrees of freedom pull-in model. *Sens. Actuators A Phys.* **2002**, *97*, 569–578.
4. Yildiz, A.; Ak, C.; Canbolat, H. New Approach to Pull-In Limit and Position Control of Electrostatic Cantilever within the Pull-in Limit. In *Electrostatics*; InTech: Rijeka, Croatia, 2007.
5. Saha, S.C.; Hanke, U.; Jensen, G.U.; Sœther, T. Modeling of spring constant and pull-down voltage of non uniform RF MEMS cantilever. In Proceedings of the 2006 IEEE International Behavioral Modeling and Simulation Workshop, BMAS 2006, San Jose, CA, USA, 14–15 September 2006.
6. Chowdhury, S.; Ahmadi, M.; Miller, W.C. A closed-form model for the pull-in voltage of electrostatically actuated cantilever beams. *J. Micromech. Microeng.* **2005**, *15*, 756–763.
7. Sadeghian, H.; Rezazadeh, G.; Osterberg, P. Application of the Generalized Differential Quadrature Method to the Study of Pull-in Phenomena of MEMS Switches. *J. Microelectromech. Syst.* **2007**, *16*, 1334–1340.
8. Pamidighantam, S.; Puers, R.; Baert, K.; Tilmans, H.A.C. Pull-in voltage analysis of electrostatically actuated beam structures with fixed-fixed and fixed-free end conditions. *J. Micromech. Microeng.* **2002**, *12*, 458–464.

9. Gere, J.; Goodno, B. *Mechanics of Materials*, 7nd ed.; CL-Engineering: Stanford, CA, USA, 2008.
10. Kokorian, J.; Buja, F.; Staufer, U.; van Spengen, W.M. An optical in-plane displacement measurement technique with sub-nanometer accuracy based on curve-fitting. In Proceedings of the 2014 IEEE 27th International Conference on Micro Electro Mechanical Systems (MEMS), San Francisco, CA, USA, 26–30 January 2014; pp. 580–583.
11. Crisfield, M.A. *Non-Linear Finite Element Analysis of Solids and Structures*; Wiley: West Sussex, UK, 1997; Volume 2.
12. Pandey, A.K.; Pratap, R. Effect of flexural modes on squeeze film damping in MEMS cantilever resonators. *J. Micromech. Microeng.* **2007**, *17*, 2475–2484.
